# Identification of MltG as a Prc Protease Substrate Whose Dysregulation Contributes to the Conditional Growth Defect of Prc-Deficient *Escherichia coli*

**DOI:** 10.3389/fmicb.2020.02000

**Published:** 2020-08-27

**Authors:** Po-Chuen Hsu, Chien-Sheng Chen, Shuying Wang, Masayuki Hashimoto, Wen-Chun Huang, Ching-Hao Teng

**Affiliations:** ^1^Institute of Molecular Medicine, College of Medicine, National Cheng Kung University, Tainan, Taiwan; ^2^Department of Food Safety/Hygiene and Risk Management, College of Medicine, National Cheng Kung University, Tainan, Taiwan; ^3^Department of Microbiology and Immunology, College of Medicine, National Cheng Kung University, Tainan, Taiwan; ^4^Institute of Basic Medical Sciences, College of Medicine, National Cheng Kung University, Tainan, Taiwan; ^5^Center of Infectious Disease and Signaling Research, National Cheng Kung University, Tainan, Taiwan; ^6^Department of Biotechnology and Bioindustry Sciences, College of Bioscience and Biotechnology, National Cheng Kung University, Tainan, Taiwan

**Keywords:** Prc, Tsp, MltG, YceG, MepS, Spr, protease, peptidoglycan

## Abstract

Microbial proteases play pivotal roles in many aspects of bacterial physiological processes. Because a protease exerts its biological function by proteolytically regulating its substrates, the identification and characterization of the physiological substrates of a protease advance our understanding of the biological roles of the protease. Prc (also named Tsp) is an *Escherichia coli* periplasmic protease thought to be indispensable for *E. coli* to survive under low osmolality at 42°C. The accumulation of the Prc substrate MepS due to Prc deficiency contributes to the conditional growth defect. Because preventing MepS accumulation only partially restored the growth of Prc-deficient *E. coli*, we hypothesized that other unidentified Prc substrates intracellularly accumulate due to Prc deficiency and contribute to the conditional growth defect. To identify previously undiscovered substrates, 85 *E. coli* proteins able to physically interact with Prc were identified using *E. coli* proteome arrays. Ten proteins were shown to be cleavable by Prc *in vitro*. Among these candidates, MltG was able to interact with Prc in *E. coli*. Prc regulated the intracellular level of MltG, indicating that MltG is a physiological substrate of Prc. Prc deficiency induced the accumulation of MltG in the bacteria. Blocking MltG accumulation by deleting *mltG* partially restored the growth of Prc-deficient *E. coli*. In addition, Prc-deficient *E. coli* with blocked MltG and MepS expression exhibited higher growth levels than those with only the MltG or MepS expression blocked under low osmolality at 42°C, suggesting that these accumulated substrates additively contributed to the conditional growth defect. MltG is a lytic transglycosylase involved in the biogenesis of peptidoglycan (PG). In addition to MltG, the previously identified physiological Prc substrates MepS and PBP3 are involved in PG biogenesis, suggesting a potential role of Prc in regulating PG biogenesis.

## Introduction

Microbial intracellular proteases govern vital physiological processes through diverse actions, such as protein quality control and the promotion of turnover, maturation and modification, which are crucial for proper function or localization of bacterial proteins (Weichart et al., 2003; Clausen et al., 2011; Dalbey et al., 2012). A protease usually exerts its physiological function through the proteolytic regulation of its substrates in an organism. Thus, identifying and characterizing substrates of a protease will provide a fundamental understanding of the biological roles of the protease. Prc, an *Escherichia coli* periplasmic protease (also known as Tsp), is physiologically important in *E. coli* as indicated by Prc deficiency completely inhibiting bacterial growth at 42°C in a low-salt medium which is commonly referred to as a condition of combined high temperature and low osmolality (Hara et al., 1991, 1996; Bass et al., 1996; Tadokoro et al., 2004). To date, whether there are other unidentified Prc substrates that are dysregulated in Prc-deficient *E. coli* contributing to the conditional growth defect has not been fully elucidated.

A 76-kDa protein, Prc is composed of an N-terminal domain, a C-terminal catalytic domain, and a PDZ domain (Beebe et al., 2000). The active site residues of Prc, Ser-430, Asp-441 and Lys-455, are located near the C-terminus and execute the proteolytic activity of Prc. The N-terminal domain of Prc is required for its ancillary function in directly or indirectly facilitating catalytic activity (Keiler and Sauer, 1995; Beebe et al., 2000). The PDZ domain is essential for substrate recognition by recognizing and binding to the nonpolar or hydrophobic C-terminal residues of substrate proteins (Beebe et al., 2000). It has been shown that a number of natural or recombinant proteins can be cleaved by Prc *in vitro* and *in vivo*. Prc can degrade mammalian β-casein and a recombinant variant of the N-terminal λ-repressor (residues 1-102), with its C-terminal 5 residues replaced with nonpolar amino acids (Silber et al., 1992; Spiers et al., 2002). In addition, Prc can recognize and degrade proteins tagged by the small stable RNA A (*ssrA*)-encoded peptide *in vitro*, suggesting that this protease may be involved in protein quality control in *E. coli* (Karzai et al., 2000). Moreover, penicillin-binding protein 3 (PBP 3) and MepS (also named Spr) have been identified as physiological substrates of Prc in *E. coli* (Hara et al., 1989, 1991; Nagasawa et al., 1989; Singh et al., 2015; Chueh et al., 2019). PBP3 is a peptidoglycan (PG) transpeptidase involved in septal PG synthesis during cell division (Hara et al., 1991), while MepS is a PG endopeptidase involved in breaking the peptide linkage between the glycan strands of a PG (Singh et al., 2012). However, the fates of these natural substrates are different; MepS is fully degraded after being proteolyzed by Prc, while only 11 residues in the C-terminal of PBP3 are cleaved (Hara et al., 1989; Nagasawa et al., 1989).

The intracellular accumulation of MepS has been shown to contribute to the conditional growth defect of Prc-deficient *E. coli* (Singh et al., 2015). However, in this study, we found that blocking MepS accumulation only partially restored the growth of Prc-deficient *E. coli* under low osmolality at 42°C, suggesting that previously unidentified Prc substrates, in addition to MepS, may play roles in the growth defect of *prc* mutants.

To identify new Prc substrates involved in the conditional growth defect of *prc* mutants, an *E. coli* proteome microarray (Chen et al., 2008) was employed to screen for proteins able to physically interact with Prc. The *E. coli* proteome array is based on the K12 open reading frame (ORF) plasmid library constructed by Kitagawa et al. (2005), which enabled us to efficiently screen approximately 99% of the *E. coli* K12 proteins (Chen et al., 2008). The candidate proteins selected based on the screening were subjected to *in vitro* Prc proteolytic assays, gene mutation analyses, and *in vivo* functional assays. Finally, we identified a new Prc natural substrate, MltG, whose intracellular accumulation in Prc-deficient *E. coli* contributed to the conditional growth defect.

## Results

### Low Osmolality Causes a Growth Defect of Prc-Deficient *E. coli* at High Temperature

**FIGURE 1:**
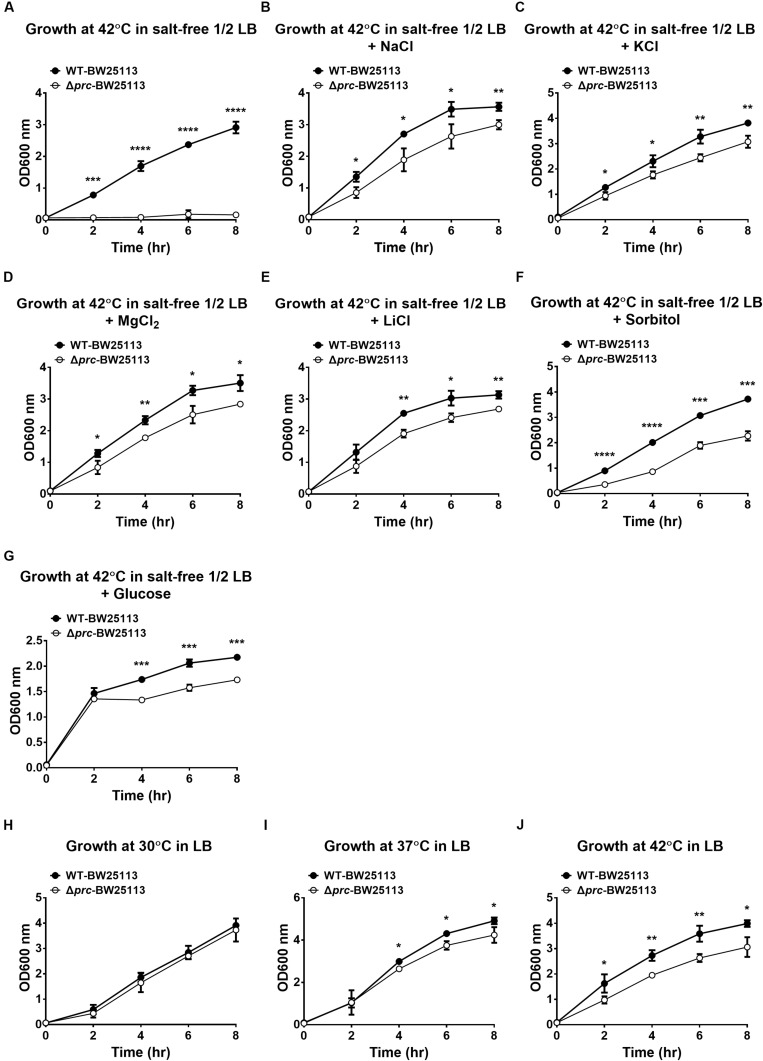
The growth of WT-BW25113 and Δ*prc*-BW25113 in media with or without osmolality adjustment. **(A–G)** The growth of the bacteria at 42°C in salt-free 1/2 LB medium without osmolality adjustment **(A)**, and with osmolality adjustment using NaCl **(B)**, KCl **(C)**, MgCl_2_ **(D)**, LiCl **(E)**, sorbitol **(F)**, and glucose **(G)**, respectively. **(H–I)** The growth of the bacteria in normal LB medium at 30°C **(H)**, 37°C **(I)**, and 42°C **(J)**. The growth of the strains was determined by measuring the values of O.D._600_ at the indicated time points using a spectrophotometer. Data are shown as the mean ± SD of three independent experiments. Asterisks denote significant differences between the O.D._600_ values of WT-BW25113 and Δ*prc*-BW25113 at the indicated time points (*, *P*-values of ≤ 0.05; **, *P*-values of ≤ 0.01; ***, *P*-values of ≤ 0.001; ****, *P*-values of ≤ 0.0001).

Previous studies have indicated that the growth of Prc-deficient *E. coli* strains is completely inhibited at 42°C in salt-free 1/2 LB medium (see Materials and Methods), referred to as low osmolality medium (Hara et al., 1991, 1996; Bass et al., 1996; Tadokoro et al., 2004). However, no direct evidence showed that the growth defect was due to the low osmolality of the medium. To confirm that low osmolality contributed to the growth defect of Prc-deficient *E. coli* at 42°C, we measured the growth of the wild-type *E. coli* K12 strain BW25113 (WT-BW25113) and its *prc* mutant (Δ*prc*-BW25113) in salt-free 1/2 LB medium with the osmolality adjusted to the level of the regular LB medium by using different solutes. The osmolality of the salt-free 1/2 LB and normal LB medium was approximately 46 mOsm/kg and 390 mOsm/kg, respectively. The strains were cultured in normal LB medium and salt-free 1/2 LB medium in which the osmolality was adjusted to approximately 390 mOsm/kg using NaCl (172.5 mM), KCl (172.5 mM), MgCl_2_ (114.1 mM), LiCl (172.7 mM), sorbitol (300 mM), and glucose (344 mM). While Δ*prc*-BW25113 did not show significant growth in salt-free 1/2 LB medium without osmolality adjustment at 42°C (Figure 1A), increased osmolality in salt-free 1/2 LB medium enabled the growth of Δ*prc*-BW25113 at high temperature (Figures 1B–G). These findings support the commonly accepted notion that osmolality is a major factor contributing to the nongrowing phenotype of Prc-deficient *E. coli* at 42°C in the salt-free 1/2 LB.

In addition, Δ*prc*-BW25113 and WT-BW25113 exhibited similar growth in LB medium at 30°C (Figure 1H), suggesting Prc-deficiency does not interfere with *E. coli* growth in LB medium at 30°C, which is consistent with the finding of a previous study (Hara et al., 1991). However, the *prc* mutant showed a significant lower level of growth than the wild-type strain in LB medium at 37°C and 42°C (Figures 1I,J). These results consistently demonstrated that temperature is also a factor that interferes with the growth of Prc-deficient *E. coli*.

### The Growth Defect of the *E. coli prc* Mutant Cannot Be Fully Restored by Relieving Intracellular *MepS* Accumulation

**FIGURE 2:**
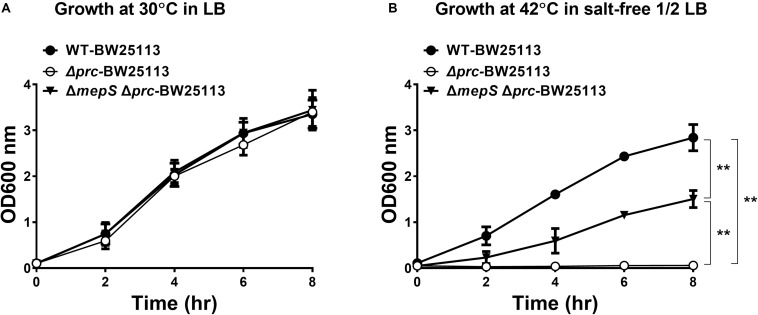
The growth of WT-BW25113, Δ*prc*-BW25113, and Δ*mepSΔprc*-BW25113. **(A)** Growth in LB medium at 30°C. **(B)** Growth in salt-free 1/2 LB medium at 42°C. Data are shown as the mean ± SD of three independent experiments. Asterisks denote significant differences between the O.D._600_ values of the indicated strains after 8 h of incubation (**, *P*-values of ≤ 0.01).

Although the growth defect of *E. coli prc* mutants under combined conditions of high temperature (42°C) and low osmolality can be alleviated by blocking intracellular MepS accumulation (Hara et al., 1996; Singh et al., 2015), it remains unclear whether MepS accumulation is the only factor contributing to the defect. To clarify this issue, we measured and compared the growth of the *prc* single mutant (Δ*prc*-BW25113), the *mepS* and *prc* double mutant (Δ*mepS*Δ*prc*-BW25113), and WT-BW25113. As shown in Figure 2A, the three strains showed similar growth in LB medium (390 mOsm/kg) at 30°C, which was the condition in which Prc deficiency did not affect bacterial growth (Figure 1H). When cultured in salt-free 1/2 LB medium (46 mOsm/kg) at 42°C, Δ*prc*-BW25113 showed no growth. The Δ*mepS*Δ*prc*-BW25113 strain, in which MepS accumulation due to Prc deficiency is blocked, exhibited a growth phenotype, as described previously (Hara et al., 1996; Singh et al., 2015). However, the growth of Δ*mepS*Δ*prc*-BW25113 was significantly lower than that of WT-BW25113 (Figure 2B). These results suggest that relief of MepS accumulation may not fully rescue the conditional growth defect caused by *prc* deletion, i.e., the growth was not recovered to the level of the wild-type strain. It is likely that the conditional growth defect caused by the *prc* deletion was the result of the dysregulation of more than one Prc substrate in the *prc* mutant. Thus, relieving the accumulation of a single substrate, MepS, can only recover the growth of the Prc-deficient strain partially but not restore the growth to the level of the WT strain. Accordingly, we hypothesized that there are possibly other unidentified Prc substrate(s) whose intracellular accumulation in the *prc* mutant also contributes to the above stated growth defect. This hypothesis led us to search for a novel Prc substrate(s) contributing to the conditional growth defect of the *prc* mutant.

### High-Throughput Screening Using a *E. coli* Proteome Array Reveals New Candidate Prc Substrates *in vitro*

**FIGURE 3:**
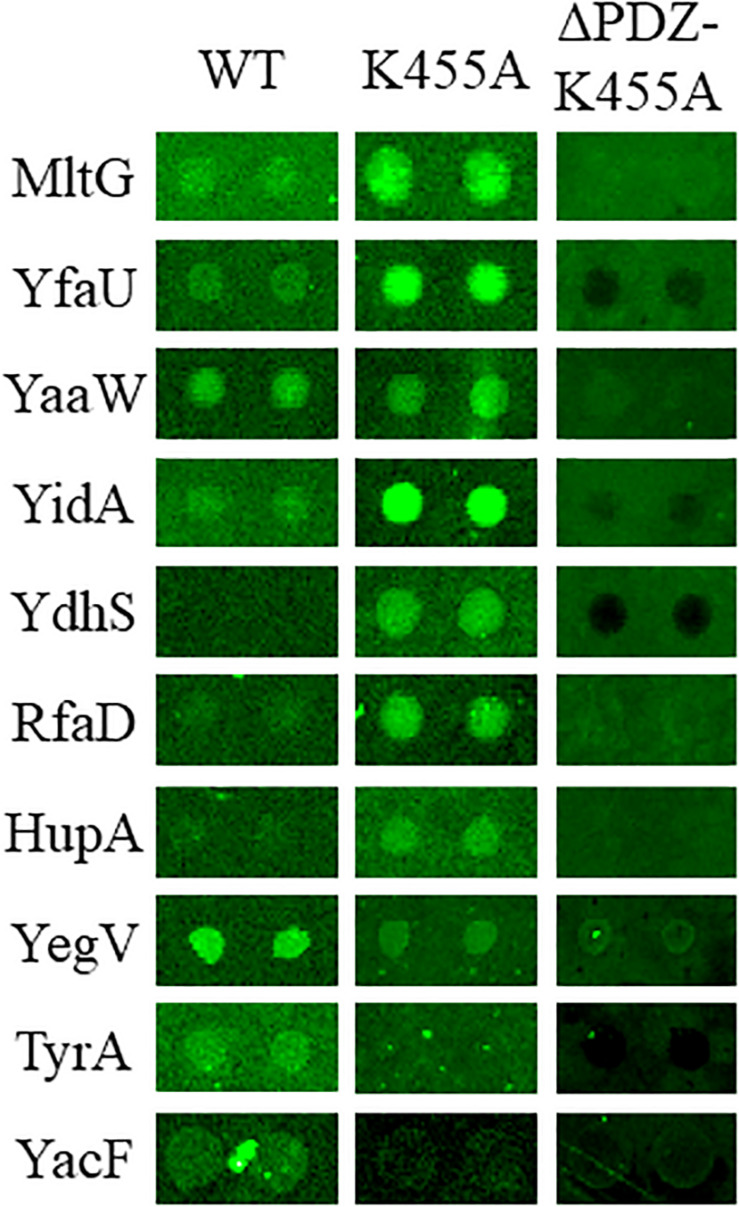
Representative images of the *E. coli* proteome array assays with the Dylight™ 549 fluorescence-labeled Prc protein variants Prc (WT), K455A-Prc (K455A), and ΔPDZ-K455A-Prc (ΔPDZ-K455A). Among these representative proteins, MltG, YfaU, YaaW, and YidA showed stronger interaction signals with Prc and K455A-Prc than with ΔPDZ-K455A-Prc. YdhS, RfaD, and HupA showed stronger interaction signals with Prc than with ΔPDZ-K455A-Prc. YegV, TyrA, and YacF showed stronger interaction signals with K455A-Prc than with ΔPDZ-K455A-Prc. The array analyses were performed in triplicate. The images of these representative protein spots were chosen from one of the triplicate experiments.

Physical interaction between a protease and its substrates is necessary for the protease to exert its proteolytic function on the substrate proteins. Thus, to identify novel substrates of Prc, we first screened for *E. coli* proteins that can physically interact with the protease. The availability of the *E. coli* K12 proteome arrays enabled us to efficiently screen 4,256 proteins to find those that interact with Prc (Chen et al., 2008). In the proteome array-based screen, fluorescently labeled Prc variants served as probes of the interactions with the proteins on the array. A potential challenge for such screening is that the substrate proteins physically associated with Prc may be degraded by the protease, leading to the loss of the fluorescence signals that would have reflected the protease-substrate interaction on the array. Thus, we generated a catalytically inactive Prc mutant (K455A-Prc) for the screening assay. The K455A-Prc protein has no catalytic activity because of the alanine substitution at Lys455 but still possesses a structure and substrate-binding ability similar to the wild-type protein (Keiler and Sauer, 1995). In addition, we generated the ΔPDZ-K455A-Prc recombinant protein, which is K455A-Prc with the deletion of the PDZ domain leading to its loss of substrate-binding ability. Fluorescently labeled Prc, K455A-Prc, and ΔPDZ-K455A-Prc were incubated with the proteome arrays. The substrates of Prc were expected to interact directly with Prc or K455A-Prc but not ΔPDZ-K455A-Prc. Using this approach, we screened for proteins that exhibited substantial signals of interaction with Prc or K455A-Prc, selecting those with signals that were significantly stronger than those with ΔPDZ-K455A-Prc (see section “MATERIALS AND METHODS”). Based on the criteria, 85 *E. coli* proteins were selected (Supplementary Table S1). Representative chip assay images of the proteins able to interact with Prc and/or K455A-Prc are shown in Figure 3. (Note: representative images of the proteins were selected and shown here because the proteins were shown to be cleavable *in vitro* in further Prc proteolytic assays).

### Ten *E. coli* Proteins Are Prc-Cleavable According to a Systematic Proteolytic Assay

**FIGURE 4:**
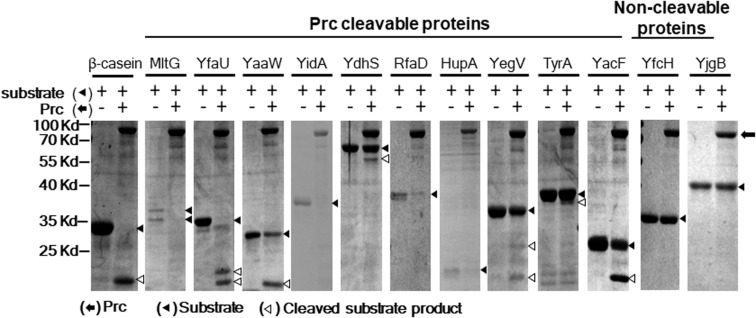
Prc proteolytic assays with *E. coli* proteins screened in the proteome assays. After incubation with or without Prc for 2.5 h, these proteins were separated by SDS–PAGE, and the protein bands on the gels were visualized by Coomassie blue staining. Among these proteins, 10 (MltG, YfaU, YaaW, YidA, YdhS, RfaD, HupA, YegV, TyrA, and YacF) were fully or partially cleaved by Prc, while the other proteins were not cleaved. YfcH and YjgB were randomly selected as representatives of the proteins that were not cleavable by Prc. β-casein, a protein known to be cleavable by Prc, served as the proteolytic positive control. Representative results of at least two independent experiments using at least two independent protein preparations are presented.

To further scrutinize whether the 85 *E. coli* proteins could be cleaved by Prc, we performed *in vitro* Prc proteolytic assays (Wang et al., 2012). The 85 N-terminally 6xHis-tagged recombinant protein candidates were overexpressed from the corresponding ORF clones in the ASKA library (Kitagawa et al., 2005). They were purified by the 96-well plate-based method developed previously (Chen et al., 2008), which is a high-throughput protocol allowing the simultaneous purification of 85 proteins in small amounts. Then, these recombinant proteins were examined by Prc proteolytic assays, with β-casein, a known Prc-cleavable protein, as a proteolytic positive control. Among these candidates, 10 of the *E. coli* proteins, MltG, YfaU, YaaW, YdhS, YidA, RfaD, HupA, YegV, TyrA, and YacF (Table 1), were shown to be fully or partially cleaved by the Prc protease *in vitro* (Figure 4), while the others were not affected by the protease. These *in vitro* cleavable proteins were potential Prc substrates in *E. coli* cells.

**TABLE 1:** The Prc-cleavable proteins.

Protein	Gene	EcoGene ID	Location	Functions
MltG	*mltG*	EG11494	IM	Endolytic murein transglycosylase
YfaU	*rhmA*	EG14083	C	2-Keto-3-deoxy-L-rhamnonate aldolase
YaaW	*yaaW*	EG14340	Unknown	Unknown
YdhS	*ydhS*	EG13953	Unknown	Unknown
YidA	*yidA*	EG11195	C	Sugar phosphatase
RfaD	*rfaD*	EG10838	C	ADP-L-glycero-D-manno-heptose-6-epimerase
HupA	*hupA*	EG10466	C	HU DNA-binding transcriptional dual regulator
YegV	*yegV*	EG14065	C	Putative sugar kinase
TyrA	*tyrA*	EG11039	C	Fused chorismate mutase/prephenate dehydrogenase
YacF	*zapD*	EG12313	C	Cell division

*IM, inner membrane; C, cytoplasm.*

### Deletion of *mltG*, the Gene of a Potential Substrate, Suppresses the Conditional Growth Defect of the *prc* Mutant

**FIGURE 5:**
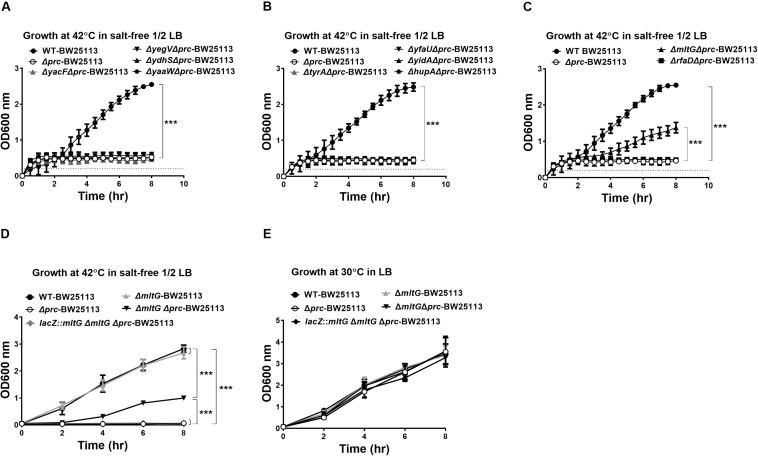
Screening for potential Prc substrates involved in the growth defect of Prc-deficient *E. coli* under low osmolality at high temperature. **(A–C)** The growth of the double mutants of the potential substrates and Prc in salt-free 1/2 LB medium at 42°C. WT-BW25113 and Δ*prc*-BW25113 served as the growth positive and negative controls, respectively. The growth of the strains was measured by a noninvasive turbidity meter, which can automatically assess multiple samples simultaneously. **(D,E)** The growth of WT-BW25113, Δ*prc*-BW25113, Δ*mltG*-BW25113, Δ*mltGΔprc*-BW25113, and *lacZ::mltG-ΔmltGΔprc*-BW25113 in salt-free 1/2 LB medium at 42°C **(D)** and in normal LB medium at 30°C **(E)**. The growth of the strains was determined by measuring the values of O.D._600_ at the indicated time points using a spectrophotometer. Data are shown as the mean ± SD of three independent experiments. Asterisks denote significant differences between the O.D._600_ values of the indicated strains after 8 h of incubation (***, *P*-values of ≤ 0.001).

The 10 proteins shown to be cleavable by Prc (Figure 4) were further investigated for their involvement in the conditional growth defect of the *E. coli prc* mutant. Based on our hypothesis that the intracellular accumulation of Prc substrates, in addition to MepS, may also contribute to the defective growth of the *prc* mutant, deletion of the genes encoding the corresponding substrates may block their intracellular accumulation in the *prc* mutant and thus suppress the defective phenotype. Accordingly, the deletion mutations of the genes encoding these Prc-cleavable proteins were introduced into Δ*prc*-BW25113. Then, the double mutants were subjected to screening for those able to grow in salt-free 1/2 LB medium at 42°C (Figures 5A–C). Among these mutants, only the Δ*mltG*Δ*prc* double mutant (Δ*mltG*Δ*prc*-BW25113) showed the ability to grow under this condition, while the other double mutants, similar to Δ*prc*-BW25113, showed no growth. Consistent with these findings, trans-complementation with a copy of *mltG* in the *lacZ* chromosomal locus of Δ*mltGΔprc*-BW25113 (the resulting strain was *lacZ::mltG-ΔmltGΔprc*-BW25113) resulted in the same growth defect as that observed for Δ*prc*-BW25113. However, the *mltG* single mutant (Δ*mltG*-BW25113) showed no significant growth defect (Figure 5D). In addition, WT-BW25113, Δ*prc*-BW25113, Δ*mltG*-BW25113, Δ*mltG*Δ*prc*-BW25113, and *lacZ::mltG-ΔmltGΔprc*-BW2511 showed similar growth in normal LB medium at 30°C (Figure 5E). These results indicated that blocking MltG expression suppresses the conditional growth defect caused by the *prc* deletion and suggest that MltG was a potential substrate responsible for the conditional growth defect of the *prc* mutant. Thus, we then focused on MltG in the following investigations.

### *In vitro* Prc Proteolytic Assays With Purified Recombinant MltG Proteins

**FIGURE 6:**
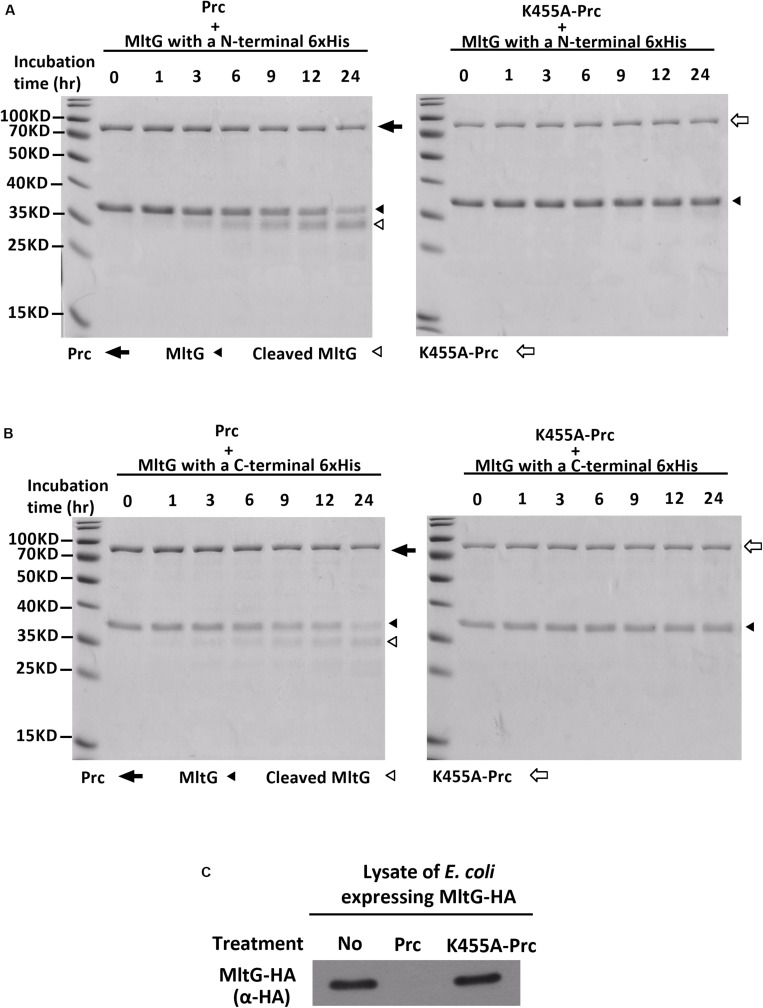
*In vitro* Prc-mediated proteolysis of MltG. **(A)** Proteolytic assays of N-terminally 6xHis_–_tagged MltG with Prc or K455A-Prc. **(B)** Proteolytic assays of C-terminally 6xHis_–_tagged MltG with Prc or K455A-Prc. For panels A and B, the Prc-MltG mixtures at the indicated time points post-incubation were subjected to SDS–PAGE and Coomassie blue staining. **(C)** The lysates of Δ*mltGΔprc*-BW25113/pBAD24-MltG-HA (Table 2), which expressed C-terminally HA-tagged MltG but not Prc, were subjected to proteolytic analyses with purified Prc or K455A-Prc. The levels of MltG in the lysates at 3 h post-incubation were determined by Western blotting with anti-HA antibodies. For **(A–C)**, representative results of two independent experiments using two independently purified proteins are presented.

MltG is a protein associated with the inner membrane. The *mltG* gene encodes the pro-MltG protein, which contains an N-terminal signal peptide (Yunck et al., 2016). The signal peptide is removed after the MltG protein is translocated to the inner membrane. However, the MltG recombinant protein used in the Prc proteolytic assays (Figure 4) still contained the signal peptide. It cannot be excluded that the presence of the signal peptide makes the recombinant protein cleavable by Prc *in vitro*. Additionally, because the recombinant protein was purified with a high-throughput method, the protein preparation may have contained other bacterial factors. To further confirm whether the pure MltG protein can be cleaved by Prc *in vitro*, we cloned and purified N-terminal signal sequence-truncated MltG recombinant proteins with a C-terminal or N-terminal 6xHis tag. To obtain pure recombinant proteins, we used Ni-affinity and subsequent size exclusion chromatography. As shown in Figures 6A,B, the recombinant MltG proteins could be cleaved by Prc regardless of the location of the 6xHis tag but not by K455A-Prc. This result demonstrates that MltG can be cleaved by Prc *in vitro* although approximately 12 h were required for the protease to cleave one-half of the purified MltG proteins. In addition, the column-purified *E. coli* proteins, YfcH and YjgB, whose high throughput preparations were shown to be noncleavable by Prc (Figure 4), showed no degradation after incubation with Prc for up to 24 h (data not shown), suggesting that Prc degrades MltG specifically under this *in vitro* condition.

We investigated whether MltG could be more efficiently cleaved by Prc in the presence of other bacterial factors. Lysates of the *E. coli* strain expressing MltG but not Prc (Δ*mltGΔprc*-BW25113/pBAD24-MltG-HA) were subjected to proteolytic analysis with purified Prc or K455A-Prc. As shown in Figure 6C, MltG was fully degraded by Prc but not K455A-Prc after 3 h of incubation. This result may suggest presence of factors *in vivo* increases the efficiency of MltG degradation by Prc, which may mimic the intracellular condition of *E. coli* cells.

### The Dysregulation and Accumulation of MltG in the *prc* Mutant Contribute to the Bacterial Growth Defect

**FIGURE 7:**
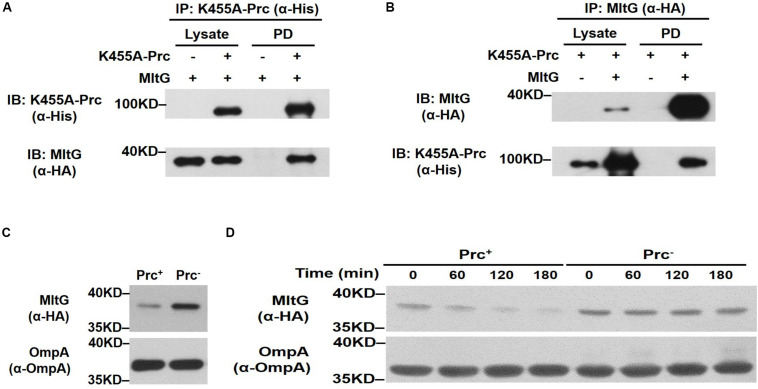
Effect of Prc on the intracellular level of MltG in *E. coli*. **(A,B)** Validation of the *in vivo* interaction between Prc and MltG. Δ*mltG*-BW25113 harboring the plasmids pTR163 (Spiers et al., 2002) and pACYC184-MltG-HA were used to coexpress K455A-Prc and HA-tagged MltG. K455A-Prc was immunoprecipitated (IP) with anti-His antibodies **(A)**, while the MltG protein was precipitated with anti-HA antibodies **(B)**. The pull-down fractions (PD) were analyzed by immunoblotting (IB) with anti-His or anti-HA antibodies. **(C)** The levels of MltG in *E. coli* strains with and without Prc. The levels of MltG in BW25113-MltG-HA (Prc^+^) (Table 2) and Δ*prc*-BW25113-MltG-HA (Prc^–^) were determined by Western blot analysis of the log-phase bacterial cultures with anti-HA antibodies. **(D)** *In vivo* degradation assays of MltG in the bacterial strains with or without Prc. The MltG levels in BW25113-MltG-HA (Prc^+^) and Δ*prc*-BW25113-MltG-HA (Prc^–^) were determined at the indicated time points after spectinomycin treatment. For **(A,B)**, representative results of three independent experiments are presented. For panels **(C,D)**, representative results of two independent experiments are presented. The outer membrane protein OmpA was used as a protein-loading control.

We further investigated whether MltG can be cleaved by Prc within bacterial cells. First, to examine whether Prc interacts with MltG *in vivo*, we performed coimmunoprecipitation assays. An MltG protein with a C-terminal HA tag was coexpressed with K455A-Prc, which contained a C-terminal 6xHis-tag (Spiers et al., 2002), in Δ*mltG*-BW25113. To precipitate K455A-Prc and the HA-tagged MltG, the lysate of the bacteria was incubated with anti-His and anti-HA antibodies, respectively, followed by antibody-antigen complex precipitation using protein G-coated magnetic beads. Immunoblot analysis of the K455A-Prc and MltG fractions pulled down with anti-HA and anti-His antibodies showed that MltG and K455A-Prc coprecipitated, respectively (Figures 7A,B). These results suggest that MltG interacts with the Prc protease in *E. coli* cells.

Second, to determine whether the presence of Prc affects the intracellular level of MltG in *E. coli*, the *E. coli* strains BW25113-MltG-HA and Δ*prc*-BW25113-MltG-HA (Table 2) were constructed by fusing the HA tag sequences to the 3′ ends of the chromosomal *mltG* genes in WT-BW25113 and Δ*prc*-BW25113, allowing the expression of HA-tagged MltG. The MltG levels in the *E. coli* strains in log phase with and without the *prc* gene were determined by Western blot analysis with anti-HA antibodies. As shown in Figure 7C, the MltG level in Δ*prc*-BW25113-MltG-HA was significantly higher than that in BW25113-MltG-HA, suggesting that Prc regulates the intracellular level of MltG and that inactivation of *prc* causes the accumulation of MltG.

**TABLE 2:** Bacterial strains used in this study.

Strains	Relevant information	Source
WT-BW25113	The wild-type *E. coli* K12 strain BW25113	Baba et al., 2006
Δ*prc*-BW25113	BW25113 with a *prc* deletion mutation	This study
Δ*mltG*-BW25113	BW25113 with a *mltG* deletion mutation	This study
Δ*mepSΔprc*-BW25113	Δ*prc*-BW25113 with an *mepS* deletion mutation	This study
Δ*mltGΔprc*-BW25113	Δ*prc*-BW25113 with a *mltG* deletion mutation	This study
Δ*yfaUΔprc*-BW25113	Δ*prc*-BW25113 with a *yfaU* deletion mutation	This study
Δ*yaaWΔprc*-BW25113	Δ*prc*-BW25113 with a *yaaW* deletion mutation	This study
Δ*ydhSΔprc*-BW25113	Δ*prc*-BW25113 with a *ydhS* deletion mutation	This study
Δ*yidAΔprc*-BW25113	Δ*prc*-BW25113 with a *yidA* deletion mutation	This study
Δ*rfaDΔprc*-BW25113	Δ*prc*-BW25113 with an *rfaD* deletion mutation	This study
Δ*hupAΔprc*-BW25113	Δ*prc*-BW25113 with a *hupA* deletion mutation	This study
Δ*yegVΔprc*-BW25113	Δ*prc*-BW25113 with a *yegV* deletion mutation	This study
Δ*tyrAΔprc*-BW25113	Δ*prc*-BW25113 with a *tyrA* deletion mutation	This study
Δ*yacFΔprc*-BW25113	Δ*prc*-BW25113 with a *yacF* deletion mutation	This study
Δ*nlpIΔmltG*-BW25113	Δ*mltG*-BW25113 with an *nlpI* deletion mutation	This study
*lacZ::mltG-ΔmltGΔprc*-BW25113	Δ*mltGΔprc*-BW25113 with an insertion of *mltG* in *lacZ*	This study
Δ*mltGΔmepSΔprc*-BW25113	Δ*mepSΔprc*-BW25113 with a *mltG* deletion	This study
BW25113-MltG-HA	BW25113 with a *mltG*-*HA* at the original *mltG* chromosomal locus	This study
Δ*prc*-BW25113-MltG-HA	BW25113-MltG-HA with a *prc* deletion mutation	This study
BW25113-MepS-3xFlag	BW25113 with a *mepS*-*3xFlag* at the original *mepS* chromosomal locus	This study
Δ*mltG*-BW25113-MepS-3xFlag	Δ*mltG*-BW25113 with a *mepS*-*3xFlag* at the original *mepS* chromosomal locus	This study
Δ*prc-*BW25113-MepS-3xFlag	Δ*prc*-BW25113 with a *mepS*-*3xFlag* at the original *mepS* chromosomal locus	This study
Δ*mltGΔprc*-BW25113-MepS-3xFlag	Δ*mltGΔprc*-BW25113 with a *mepS*-*3xFlag* at the original *mepS* chromosomal locus	This study
BW25113-MltG-HA-MepS-3xFlag	BW25113-MepS-3xFlag with a *mltG*-*HA* at the original *mltG* chromosomal locus	This study
Δ*nlpI*-BW25113-MltG-HA-MepS-3xFlag	BW25113-MltG-HA-MepS-3xFlag with a *nlpI* deletion	This study

Finally, to further investigate the basis of the Prc-mediated regulation of MltG in *E. coli*, a pulse–chase experiment (*in vivo* degradation assay) was performed after the inhibition of protein synthesis by treatment with the ribosome inhibitor spectinomycin. As shown in Figure 7D, after blocking protein synthesis, the MltG level decreased significantly in BW25113-MltG-HA within 60 min, while this protein was maintained at a similar level in Δ*prc*-BW25113-MltG-HA. This result demonstrated that the increased MltG level in the Prc-deficient strain (Figure 7C) is a consequence of enhanced posttranslational stability, suggesting that Prc regulates the intracellular level of MltG through its proteolytic function.

Taken together, these findings, in conjunction with the observation that the deletion of *mltG* can relieve the growth defect of the *E. coli prc* mutant at high temperature and low osmolality (Figures 5C,D), strongly suggest that the accumulation of MltG contributes to the growth defect in Prc-deficient *E. coli*.

### Overexpression of MltG Hinders Bacterial Growth Under Low Osmolality at High Temperature

**FIGURE 8:**
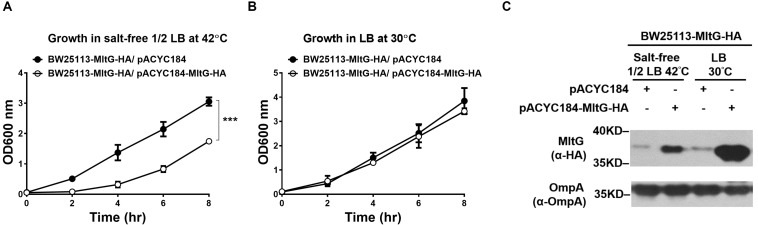
The effect of the intracellular accumulation of MltG on the growth of *E. coli*. **(A)** The growth of BW25113-MltG-HA harboring pACYC184-MltG-HA or the empty vector pACYC184 in salt-free 1/2 LB medium at 42°C. **(B)** The growth of the strains in normal LB medium at 30°C. For **(A)** and **(B)**, the data are shown as the mean ± SD of three independent experiments. Asterisks denote significant differences between the O.D._600_ values of the indicated strains after 8 h of incubation (***, *P*-values of ≤ 0.001). **(C)** The MltG levels in BW25113-MltG-HA with or without MltG overexpression under 42°C/salt-free 1/2 LB medium and 30°C/LB medium conditions. The levels of MltG were determined by Western blot analysis of the log-phase bacteria (2 h of incubation of 100-fold-diluted overnight culture) with anti-HA antibodies. The outer membrane protein OmpA was used as a protein-loading control. Representative results of two independent experiments are presented.

We further investigated whether increasing the intracellular level of MltG without the loss of Prc function can interfere with *E. coli* growth in salt-free 1/2 LB medium at 42°C. To raise the intracellular MltG level, the plasmid pACYC184-MltG-HA was introduced into BW25113-MltG-HA (BW25113-MltG-HA/pACYC184-MltG-HA) to allow overexpression of MltG. BW25 113-MltG-HA/pACYC184-MltG-HA exhibited a significantly low level of grow than did the BW25113-MltG-HA strain harboring the empty plasmid vector (BW25113-MltG-HA/pACYC184) at 42°C in salt-free 1/2 LB medium, while the strains exhibited a similar level of growth at 30°C in normal LB medium (Figures 8A,B). Additionally, as expected, BW25113-MltG-HA/pACYC184-MltG-HA showed higher levels of MltG than BW25113-MltG-HA/pACYC184 in both 42°C/salt-free 1/2 LB medium and 30°C/LB medium conditions (Figure 8C). This result suggests that increasing the intracellular level of MltG alone can interfere with bacterial growth under low osmolality at high temperature, further supporting the notion that the increased MltG level in Prc-deficient *E. coli* contributes to the conditional growth defect of the bacteria.

### MepS Accumulation in the *prc* Mutant Is Independent of MltG Accumulation

**FIGURE 9:**
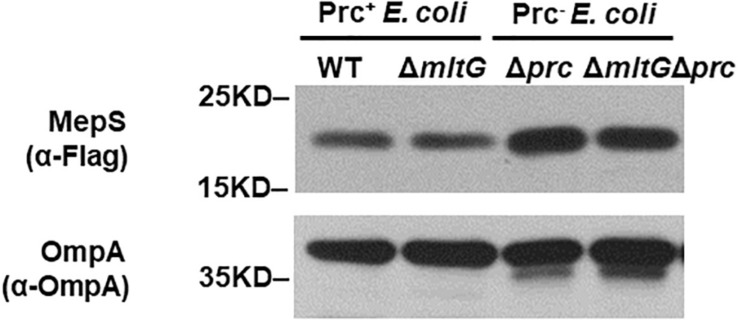
Effect of *mltG* deletion on the intracellular level of MepS. The levels of MepS in the *E. coli* strains were measured by Western blot analysis using a C-terminally 3xFLAG-tagged MepS encoded at the native chromosomal locus. A log-phase culture of each strain was collected and subjected to Western blot analysis. The outer membrane protein OmpA was used as a protein-loading control. Representative results of two independent experiments are presented. WT: BW25113-MepS-3xFlag; Δ*mltG*: Δ*mltG*-BW25113-MepS-3xFlag; Δ*prc*: Δ*prc*-BW25113-MepS-3xFlag; and Δ*mltGΔprc*: Δ*mltGΔprc*-BW25113-MepS-3xFlag (Table 2).

To clarify whether the accumulation of MltG affects the accumulation of MepS in the *prc* mutant, we compared the MepS levels in *E. coli* strains without *mltG* to those in the strains with *mltG*, in the backgrounds with and without *prc*. As shown in Figure 9, deletion of *mltG* did not aggravate or alleviate the levels of MepS in either the strains with Prc^+^ or Prc^–^ backgrounds. Additionally, consistent with a previous study (Singh et al., 2015), the MepS levels in the strains without *prc* were significantly higher than those in the strains with *prc*. These results demonstrate that relieving MltG accumulation through the deletion of *mltG* does not affect the MepS level in the *prc* mutant. Thus, it is reasonable to conclude that the contribution of MltG accumulation to the conditional growth defect of the *prc* mutant does not work through affecting the MepS level.

### The Interaction Between Prc and MltG Does Not Require the Adaptor Protein NlpI

**FIGURE 10:**
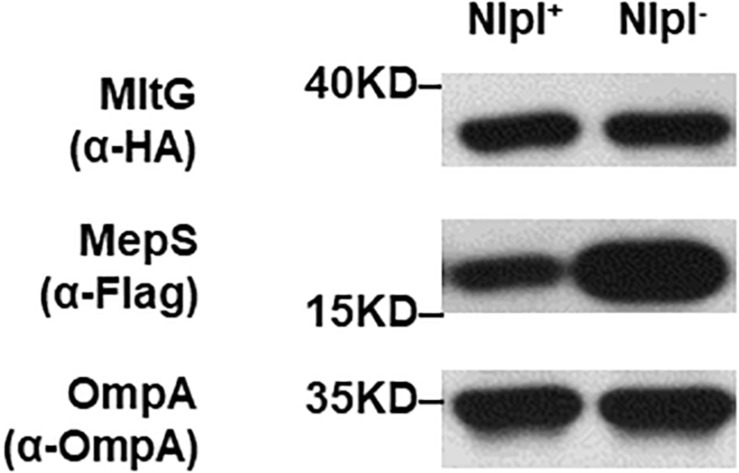
MltG levels in *nlpI* mutant strains. The levels of MltG in BW25113-MltG-HA-MepS-3xFlag and Δ*nlpI*-BW25113-MltG-HA- MepS-3xFlag (Table 2) were measured by Western blotting with anti-HA antibodies. The levels of MepS were measured with anti-Flag antibodies. The outer membrane protein OmpA was used as a protein-loading control. Representative results of two independent experiments are presented. NlpI^+^: BW25113-MltG-HA-MepS-3xFlag; NlpI^–^: Δ*nlpI*-BW25113-MltG-HA- MepS-3xFlag.

The interaction between the Prc protease and the MepS substrate requires the outer membrane-anchored protein NlpI as an adaptor (Singh et al., 2015). Deletion of *nlpI* also results in the intracellular accumulation of MepS in *E. coli* because the intracellular interaction of Prc and MepS is abolished. We further investigated whether NlpI is also involved in the Prc-mediated proteolytic regulation of MltG. We measured and compared the levels of MltG in the BW25113 strains with or without *nlpI* deleted (BW25113-MltG-HA-MepS-3xFlag and Δ*nlpI*-BW25113-MltG-HA-MepS-3xFlag; Table 2). As shown in Figure 10, deletion of *nlpI* did not increase the level of MltG in *E. coli*. However, the *nlpI* deletion caused MepS accumulation, which is consistent with the finding from a previous study (Singh et al., 2015). This result suggests that, in contrast to the MepS-Prc interaction, the interaction between MltG and Prc does not require NlpI as an adaptor (Figure 10).

### The Regulation of MltG by Prc Is Growth Phase-Independent

**FIGURE 11:**
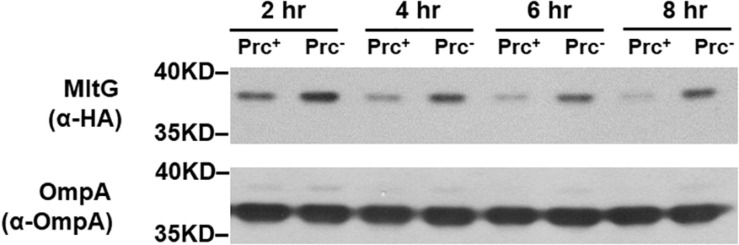
Levels of MltG in *E. coli* strains with or without Prc in cultures of different growth stages. Western blot analysis of HA-tagged MltG in BW25113-MltG-HA and Δ*prc-*BW25113-MitG-HA (Table 2). Prc^+^, BW25113-MltG-HA; Prc^+^, Δ*prc-*BW25113-MitG-HA. The outer membrane protein OmpA was used as a protein-loading control. Representative results of two independent experiments are presented.

To investigate whether MltG regulation by Prc is growth phase-dependent, we measured the MitG levels in *E. coli* strains with or without Prc after different growth periods. As shown in Figure 11, the level of MltG in the Prc-expressing strain BW25113-MltG-HA was lower than that in the Prc-deficient strain Δ*prc-*BW25113-MltG-HA (Table 2) in each of the indicated cultivation time of 2 h to 8 h (from the log phase to the stationary phase). These results suggest that Prc regulates the level of MltG in all bacterial growth stages.

### The Growth of WT-BW25113, Δ*mepSΔmltGΔprc*-BW25113, Δ*mltGΔprc*-BW25113, Δ*mepSΔprc*-BW25113, and Δ*prc*-BW25113 in Salt-Free 1/2 LB Medium at 42°C

**FIGURE 12:**
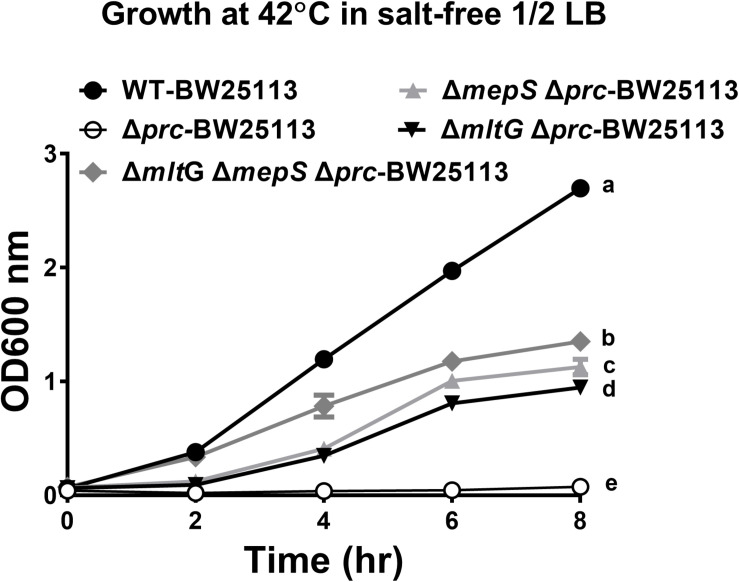
The growth of WT-BW25113, Δ*mepSΔmltGΔprc*-BW25113, Δ*mltGΔprc*-BW25113, Δ*mepSΔprc*-BW25113, and Δ*prc*-BW25113 in salt-free 1/2 LB medium at 42°C. The growth curves of the strains were determined by measuring the values of O.D._600_ at the indicated time points using a spectrophotometer. Data are shown as the mean ± SD of three independent experiments. Different letters (a,b,c,d, and e) indicate significances (*P* ≤ 0.05) between groups after 8 h of incubation. The data were analyzed by One-way ANOVA (Tukey’s multiple comparisons test).

To investigate whether blocking the accumulation of MepS and MltG restores the growth of Prc-deficient *E. coli*, the growth of Δ*mepSΔmltGΔprc*-BW25113, Δ*mltGΔprc*-BW25113, Δ*mepSΔprc*-BW25113, Δ*prc*-BW25113, and WT-BW25113 in salt-free 1/2 LB medium at 42°C was measured. As shown in Figure 12, Δ*mepSΔmltGΔprc*-BW25113 exhibited a significantly higher level of growth than did Δ*mltGΔprc*-BW25113 or Δ*mepSΔprc*-BW25113 but did not reach the same growth level as WT-BW25113. These findings demonstrate that simultaneously blocking MltG and MepS accumulation enhances the growth of Prc-deficient *E. coli* to a greater extent than did blocking the accumulation of either of these proteins, thus suggesting that the accumulation of MltG and MepS addictively contributes to the conditional growth defect caused by Prc deficiency.

## Discussion

In the present study, we identified a new Prc physiological substrate, MltG, whose intracellular level was increased by Prc deficiency. This dysregulated level was responsible for the growth defect of Prc-deficient *E. coli* under low osmolality at high temperature. The outcome of MltG dysregulation due to Prc deficiency was similar to that of the dysregulation of MepS, a previously identified substrate. The intracellular accumulation of MltG and MepS additively contributed to the conditional growth defect. MltG is a lytic transglycosylase (LT) involved in PG synthesis (Yunck et al., 2016). In addition to MltG, MepS and PBP3 (another previously identified substrate) are also involved in PG synthesis (Sauvage et al., 2008; Singh et al., 2012). Taking into account the functions of these substrates, Prc may play a potential role in regulating PG synthesis.

MltG is anchored to the inner membrane, with its functional domain exposed to the periplasm (Yunck et al., 2016). The LT activity of MltG is able to cleave nascent glycan strands of the PG meshwork and therefore terminate strand elongation during the course of PG polymerization (Yunck et al., 2016). It has been shown that a specific intracellular level of MltG is required for maintaining the integrity of the PG meshwork of bacteria. Deficiency of MltG alters the glycan strand length of PG in *E. coli*. On the other hand, a high level of MltG overexpression induces morphological defects and cell lysis, suggesting that a high level of MltG may damage the PG meshwork (Yunck et al., 2016).

The unfettered LT activity of MltG may be responsible for the conditional growth defect of Prc-deficient *E. coli*. PG is an essential structure that fortifies the cytoplasmic membrane against osmotic pressure. Thus, the cell wall is a key structure to withstand outwardly directed osmotic pressure (turgor pressure), thereby preventing cell lysis. Turgor pressure is determined by the difference in the osmolality of the cytoplasm and that of the external medium and can be calculated with the Morse equation, *p* = RT (*C*_in_ – *C*_out_), where *C*_in_ is the osmolality of the cytoplasm, *C*_out_ is the osmolality of the external medium, R is the gas constant, and T is the temperature. When bacteria are transferred to a growth medium of lower osmolality, the turgor pressure increases. Then, the bacterial cells actively reduce cytoplasmic osmolality to downregulate the raised turgor pressure to adapt to the new environment (Record et al., 1998; Cayley et al., 2000). The PG damage caused by MltG accumulation may cripple the ability of the cell wall to withstand the increased turgor pressure in Prc-deficient *E. coli* before the active cytoplasmic osmolality modification takes place, thus resulting in the bacterial growth defect. In addition, it has been shown that in phosphate minimal medium the active reduction of cytoplasmic osmolality does not fully eliminate the turgor pressure rise caused by an external osmolality decrease (Record et al., 1998; Cayley et al., 2000). It is also likely that when *E. coli* are transferred from normal LB medium to salt-free 1/2 LB medium, the bacterial turgor pressure still rises in spite of the active modification of cytoplasmic osmolality, and the pressure increase is high enough to impair the growth of the Prc-deficient bacteria. Furthermore, based on the Morse equation, the turgor pressure increases as the temperature increases. Therefore, impaired growth of Prc-deficient *E. coli* in LB medium was observed at 42°C and 37°C but not at 30°C (Figure 1H–J).

Prc regulates the intracellular levels of MltG and MepS in different ways. The Prc-mediated degradation of MepS requires the adaptor protein NlpI to facilitate the protease-substrate interaction *in vivo* (Singh et al., 2015; Su et al., 2017). NlpI, however, is not involved in Prc-mediated MltG degradation (Figure 10). There may be other accessory bacterial factors facilitating the Prc-MltG interaction in *E. coli*. This prospect was indicated by Prc-mediated MltG degradation being more efficient in conditions with various bacterial factors, such as in *E. coli* cells (Figure 7D) and in *E. coli* lysates (Figure 6C), than in the condition using only the column-purified MltG and Prc without other bacterial factors (Figures 6A,B). In addition, the MltG protein purified with the high-throughput method was found to be efficiently degraded by Prc *in vitro* (Figure 4). There may be some residual accessory factors in the protein sample that facilitated the MltG degradation by Prc.

In addition to MltG, nine other *E. coli* proteins were shown to be able to interact with Prc or/and K455A-Prc (Figure 3) and were cleavable by Prc *in vitro* (Figure 4 and Table 1). Most of these proteins were likely not physiological substrates of Prc, as indicated by their cellular localization. As Prc is a periplasmic protease, its physiological substrates are expected to be located or at least partially exposed to the periplasmic space. However, of the nine proteins, seven, YfaU, YidA, RfaD, HupA, YegV, TyrA, and YacF, are cytoplasmic proteins (Lopez-Campistrous et al., 2005; Ishihama et al., 2008; Rea et al., 2008; Diaz-Mejia et al., 2009), suggesting that they may not be able to interact with Prc *in vivo*. Although the localization of two proteins, YaaW and YdhS, remains unknown, PSORTb software scoring showed that they are likely to be cytoplasmic proteins.

Even if all of the nine proteins are not physiological substrates, further understanding of their interaction with Prc would provide insights into how Prc recognizes and exerts its proteolytic function on substrates. However, it is worth noting that the interactions of the nine proteins with Prc were demonstrated by the high-throughput method, which may have retained some concomitant bacterial factors, as described above (Figures 3, 4). The possible involvement of certain unknown residual factors in the interaction with Prc cannot be excluded.

It has been reported that Prc preferentially recognizes and thus degrades proteins bearing a nonpolar C-terminal tail (Silber et al., 1992; Spiers et al., 2002). However, the C-termini of MltG, MepS, and PBP3 do not appear to bear this feature and, in fact, contain a substantial portion of polar residues (Supplementary Table S2). This finding suggests that a nonpolar C-terminal region may not be necessary for substrate recognition by Prc. Prc may recognize substrates without a nonpolar C-terminus, probably through the assistance of other bacterial factors, such as the adaptor NlpI for MepS and potential accessory bacterial factors for MltG. Alternatively, Prc may directly recognize regions of substrates not in the C-terminus. Further investigation will be required to understand how Prc recognizes its substrates.

Prc deficiency decreases the abilities of extraintestinal pathogenic *E. coli* (ExPEC) to cause bacteremia and urinary tract infections (UTIs) (Wang et al., 2012; Huang et al., 2020). The accumulation of MepS (Spr) contributes to the impaired ability of Prc-deficient ExPEC to colonize urinary tracts. It is worth further investigation to determine whether the dysregulation of MltG due to Prc deficiency is involved in the impaired ExPEC virulence in bacteremia and UTIs.

In summary, we identified MltG as a new physiological substrate of the periplasmic protease Prc. Prc deficiency induced MltG intracellular accumulation, which was responsible for the growth defect of Prc-deficient *E. coli* under low osmolality and high temperature. MltG is an LT involved in PG biogenesis. Increased MltG LT activity may impair the PG structure and attenuate the growth of Prc-deficient *E. coli* in a hypotonic medium at high temperature. Since the biological function of a protease is defined by its substrates, in addition to MltG, two other Prc substrates, MepS and PBP3, are also involved in PG synthesis (Sauvage et al., 2008; Singh et al., 2012). The functions of these substrates collectively suggest a potential role of Prc in regulating PG biogenesis.

## Materials and Methods

### Bacterial Strains and Culture Conditions

The bacterial strains and plasmids used in this study are listed in Tables 2, 3. All the strains were derivatives of BW25113 (Baba et al., 2006) unless otherwise indicated. The bacteria were grown in 5 ml of Luria-Bertani broth (LB) (1% tryptone, 0.5% yeast extract, and 1% NaCl) in test tubes at 37°C with shaking at 200 rpm unless otherwise indicated. To investigate bacterial growth in 30°C/LB medium and 42°C/salt-free 1/2 LB medium conditions with or without osmolality adjustment, Overnight cultures of bacteria were adjusted to an optical density (O.D._600_) of 4, transferred to fresh LB or salt-free 1/2 LB medium (0.5% tryptone and 0.25% yeast extract) (Hara et al., 1991) at a ratio of 1:100, and cultured at the indicated temperature.

**TABLE 3:** Plasmids used in this study.

Plasmid	Relevant information	References
**pTR147**	pTRc99A harboring a sequence encoding the C-terminally His_6_-tagged Prc	Spiers et al., 2002
**pTR163**	pTRc99A harboring a sequence encoding the C-terminally His_6_-tagged K455A-Prc	Spiers et al., 2002
**pTR163-**Δ**PDZ**	pTRc99A harboring a sequence encoding the C-terminally His_6_-tagged ΔPDZ-K455A-Prc variant	This study
**pBAD24**	A plasmid vector	Guzman et al., 1995
**pBAD24-MltG-HA**	pBAD24 harboring a sequence encoding the C-terminally HA-tagged MltG	This study
**pBAD24-MltG-His**	pBAD24 harboring a sequence encoding the C-terminally His_6_-tagged MltG with a signal sequence truncation	This study
**pBAD24-His-MltG**	pBAD24 harboring a sequence encoding the N-terminally His_6_-tagged MltG with a signal sequence truncation	This study
**pACYC184**	A plasmid vector	Chang and Cohen, 1978
**pACYC184-MltG-HA**	pACYC184 harboring a sequence encoding a C-terminally HA-tagged MltG	This study
**pCA3 × FLAG**	A plasmid harboring a sequence encoding a 3 × FLAG	Yamamoto et al., 2008
**pKD3**	A plasmid harboring a chloramphenicol resistance cassette	Datsenko and Wanner, 2000

### Mutant Construction

*Escherichia coli* mutants were constructed by the λ-red-recombinase-based method or P1 phage transduction as described previously (Datsenko and Wanner, 2000; Thomason et al., 2007; Wang et al., 2012). The description of the construction of the mutants and plasmids used in this study are described in Supplementary Text S1. The primers used to construct the mutants and plasmids are listed in Supplementary Table S3.

### Proteome Microarray Construction

The construction of the *E. coli* proteome chip was based on procedures described previously (Chen et al., 2008). Briefly, the *E. coli* K12 open reading frame (ORF) clone library (ASKA library), which was constructed by Dr. Mori and his colleagues and harbors 4,256 *E. coli* genes (Kitagawa et al., 2005), was used to overexpress *E. coli* proteins. Because the N-termini of the proteins were fused with a 6xHis tag, they were purified with Ni-NTA resin (Qiagen). The purified proteins were spotted in duplicate on Full Moon slides (Full Moon BioSystems) by using a ChipWriter Pro (Bio-Rad) with 48 pins. The printed microarrays were stored at −80°C until further use.

### *E. coli* Proteome Array Assays

To fluorescently label the protein probes (Prc, K455A-Prc, and ΔPDZ-K455A-Prc), an amine-reactive fluorescence dye, Dylight™ 549 NHS Ester (Thermo Scientific), which contains *N*-hydroxysuccinimide (NHS) esters able to covalently bind to amines of proteins, was utilized to label the protein probes following the manufacturer’s instructions. The maximum excitation/emission wavelength of Dylight™ 549 is 562 nm/576 nm, which is spectrally similar to Cy3.

The arrays were blocked with 1% bovine serum albumin (BSA) (Sigma-Aldrich Co.) in Tris-buffered saline (TBS; 137 mM NaCl, 2.7 mM KCl, 24.8 mM Tris-base, pH 7.5) for 5 min at room temperature (RT) to reduce nonspecific binding. The chips were probed with 80 μ of 1 μM Dylight™ 549-labeled Prc, K455A-Prc, or ΔPDZ-K455A-Prc in phosphate-buffered saline (PBS; 137 mM NaCl, 2.7 mM KCl, 10 mM Na_2_HPO_4_, 1.8 mM KH_2_PO_4_, pH 7.4) containing 1% BSA in a hybridization chamber with shaking at RT for 1 h. After incubation, the chips were washed 3 times with Tris-buffered saline-Tween 20 (TBS-T; TBS containing 0.05% Tween 20, pH 7.5). After the final wash, the chips were dried by centrifugation at 201 × *g* and then scanned by a microarray scanner (GenePix^®^ 4000B, Axon Instruments). Proteins binding to the fluorescently labeled Prc variants were detected at an excitation wavelength of 532 nm and emission wavelength of 570 nm.

### Bioinformatics Analysis of the Proteome Array Assays

The protein microarray experiments were done in triplicate. The results of the triplicate experiments were combined for bioinformatics analyses. Since there are duplicate spots for each protein on one array, there were 6 fluorescence readouts for each proteins. To analyze the images of the chip assay results, GenePix Pro 6.0 was used to align each protein spot and export all the imaged signals to text files. To adjust for probing and scanning procedure variations, inter-array signal intensities were quantile normalized (Bolstad et al., 2003). Then, ProCAT (Zhu et al., 2006) was applied to normalize (intra-array) the signals. The positive hits for Prc and K455A-Prc binding were selected based on a local cutoff, which was arbitrary defined as two standard deviation above the signal mean of for each spot. From the high hit candidates, we further selected for proteins whose interaction with Prc or K455A-Prc was significantly higher than ΔPDZ-K455A-Prc. Thus the proteins whose corresponding spots showed significantly higher levels of signals of interaction with Prc or K455A-Prc than those with ΔPDZ-K455A-Prc (*p* < 0.01, Student’s *t*-test) were selected (Supplementary Table S4).

### Protein Preparation

In the systematic Prc proteolytic assay with the proteins selected from the *E. coli* proteome array assay, the proteins were expressed from the ASKA library and purified using the 96-well-based high-throughput method developed by Chen et al. (2008). Finally, the proteins purified from 800 μl of IPTG-induced *E. coli* cultures were eluted from Ni-NTA resins (Qiagen) in 50 μl of elution buffer (50 mM NaH_2_PO_4_, 150 mM NaCl, 25% glycerol, 250 mM imidazole and 0.01% Triton X-100, pH 7.5) (Chen et al., 2008).

Prc, K455A-Prc, and ΔPDZ-K455A-Prc were purified from DH5α strains harboring pTR147, pTR163, and pTR163-ΔPDZ, respectively. Expression of these 6xHis-tagged proteins was induced by adding 0.1 mM IPTG to the culture when the cells reached an O.D._600_ of 0.6, and the culture was incubated at 30°C for another 5 h. The cells were centrifuged at 6000 × g for 20 min, resuspended in buffer A (20 mM Tris–HCl and 200 mM NaCl, pH 7.5) and disrupted by French press as described previously (Teng et al., 2010). The supernatant was loaded onto a Ni-NTA column (GE Healthcare), and the unbound proteins were washed away with 60 mM imidazole in buffer A. The proteins were eluted with 300 mM imidazole in buffer A. Fractions containing the proteins were pooled and further concentrated using a 50-kDa centrifugal membrane filter (Millipore). In this process, the solutions were dialyzed with buffer A to remove imidazole.

The C-terminally and N-terminally 6xHis-tagged MltG proteins were purified from DH5α strains harboring pBAD24-MltG-His and pBAD24-His-MltG, respectively. The expression of these MltG proteins was induced by adding 0.1 mM arabinose to the culture after the cells had reached an O.D._600_ of 0.6, and the culture was further incubated at 30°C for 5 h. Then, the cells were subjected to the protein purification process using a Ni-NTA column, as described above. The fractions containing the proteins were pooled and further purified by size-exclusion chromatography on a Superdex™ 75 column (GE Healthcare). Subsequently, the proteins were dialyzed in buffer B (20 mM Tris–HCl, 150 mM NaCl, 0.5 mM EDTA, 5% glycerol, and 3 mM DTT, pH 7.5). The purified proteins were stored at 4°C until further use.

### Prc Proteolytic Assay

The assays were performed as described previously with some modification (Wang et al., 2012). For the assays with the proteins purified with the 96-well plate-based method (Figure 4), 10 μl of the purified proteins (see section “Protein Preparation”) were incubated with 2 μg of Prc, which was dissolved in 5 μl of buffer A, at 37°C for 2.5 h, while 6 μg of β-casein was utilized as a degradation-positive control. After the proteolytic assays, the protein mixtures were subjected to SDS–PAGE analyses with 12% polyacrylamide gels, and the proteins were visualized by staining with Coomassie brilliant blue.

For the assays with the recombinant MltG proteins purified by Ni affinity and size exclusion chromatography (Figures 6A,B), 3 μg of the recombinant MltG proteins were incubated with 2 μg of Prc or K455A-Prc in 35 μl of buffer A at 37°C for the indicated time period. Then, the protein mixtures were subjected to SDS–PAGE analyses with 15% polyacrylamide gels. The protein bands on the gels were visualized by staining with Coomassie Brilliant Blue.

For the assays with *E. coli* lysates (Figure 6C), 20 ml of the of Δ*prc*-BW25113/pBAD24-MltG-HA that had been cultured overnight in LB medium with 0.2% L-arabinose was harvested by centrifugation, resuspended in 2 ml of buffer A, and subjected to French press disruption (Teng et al., 2010). The resulting bacterial lysate was diluted 10-fold with buffer A, and 5 μl of the diluted lysate was incubated with 2 μg of Prc or K455A-Prc in 15 μl of buffer A at 37°C for 3 h. Then, the samples were subjected to Western blotting with an anti-HA antibody (Sigma-Aldrich) to detect HA-tagged MltG.

### Coimmunoprecipitation Assays

The BW25113 strain harboring both pTR163 and pACYC184- MltG-HA or one of the plasmids was cultured overnight and then was transferred to fresh LB at a ratio of 1:100 and cultured for 2.5 h at 37°C. Then, to induce the expression of the 6xHis-tagged K455A-Prc, IPTG was added to the culture to a final concentration of 0.1 mM. After 5 h of incubation at 30°C, the bacteria in 50 ml of this culture were harvested by centrifugation and resuspended in 5 ml of buffer A (20 mM Tris–HCl and 200 mM NaCl, pH 7.5) containing 2% (v/v) Triton X-100 and 250 μg/ml lysozyme. The bacterial suspension was incubated on ice for 30 min, and the bacteria were disrupted by sonication. Unbroken cells and cell debris were removed by centrifugation at 20,000 × *g* for 25 min at 4°C. The protein concentrations of the resulting supernatants were determined using a Pierce™ BCA protein assay kit (Thermo Fisher Scientific, Carlsbad, CA, United States) and adjusted to 1 mg/ml using buffer A with 2% Triton X-100. For the pull-down assays, 1000 μl of the resulting protein solution was incubated with 1 μg (0.5 μl) of anti-His antibody (Sigma-Aldrich) or 3 μg (0.5 μl) of anti-HA antibody (Sigma-Aldrich) for 2 h at 4°C with gentle rocking in an Eppendorf tube. To precipitate the antibody-bound proteins, 5 μl of magnetic Dynabeads™ protein G (Thermo Fisher Scientific) was added to the solution. The solution was incubated for an additional 1 h at 4°C with gentle rocking, and the beads were precipitated by placing the tube on a magnet for 1 min. The beads were washed three times with 1 ml of buffer A containing 2% Triton X-100 and resuspended in 20 μl of SDS–PAGE sample buffer, followed by 10 min of incubation at 100°C. After precipitating the magnetic beads, the supernatant was subjected to Western blot analysis with anti-His and anti-HA antibodies to detect the presence of K455A-Prc and MltG-HA.

### *In vivo* Degradation Assays

To measure whether Prc degrades MltG in *E. coli* cells, an *in vivo* assay of MltG degradation was performed as previously described with some modification (Singh et al., 2015). The overnight culture (200 μl) consisting of MltG-HA-BW25113 or MltG-HA-Δ*prc*-BW25113 was inoculated into 20 ml of fresh LB and incubated for 4 h at 37°C, and then, spectinomycin was added at a concentration of 300 μg/mL to block translation in the cells. Then, 2-ml aliquots of the culture were incubated at 37°C. At the indicated time points, one aliquot of the sample was subjected to Western blot analysis with anti-HA antibodies to determine the amount of MltG in the bacteria. The outer membrane protein OmpA was used as a protein-loading control, which was measure with a mouse anti-OmpA serum described previously (Huang et al., 2020).

### Measurement of Osmolality

The osmolality of media was measured with the Micro-Digital i-Osmometer Type 7iM (Vogel Loser, Germany) by using freezing point depression (Pena-Verdeal et al., 2015). The osmolality was expressed as milliosmoles per liter per kilogram (mOsm/kg).

### Statistical Analysis

Student’s *t*-test was used to analyze the results of bacterial growth experiments, except that of the growth experiment of Δ*mepSΔmltGΔprc*-BW25113, Δ*mltGΔprc*-BW25113, Δ*mepSΔprc*-BW25113, Δ*prc*-BW25113, and WT-BW25113 in salt-free 1/2LB medium at 42°C was measured, which was analyzed by One-way ANOVA (Tukey’s multiple comparisons test).

## Data Availability Statement

All datasets generated for this study are included in the article/Supplementary Material.

## Author Contributions

P-CH carried out the experiments in this study. SW, C-SC, and C-HT contributed to the study conception, planning experiments, and data analysis and interpretation. MH and W-CH participated in the result discussion and technical support. All authors contributed to the article and approved the submitted version.

## Conflict of Interest

The authors declare that the research was conducted in the absence of any commercial or financial relationships that could be construed as a potential conflict of interest.
