# Supplementary material for: Identification of MltG as a Prc Protease Substrate Whose Dysregulation Contributes to the Conditional Growth Defect of Prc-Deficient Escherichia coli
Source: Front Microbiol. 2020 Aug 27;11:2000. doi: 10.3389/fmicb.2020.02000 (PMC7481392; doi:10.3389/fmicb.2020.02000)
Supplement: Supplementary file 1 [file Data_Sheet_1.docx]

**Supplementary data**

**Text S1**

**Supplementary Experimental Procedures**

**Mutant construction**

The *prc* single mutant of BW25113 was constructed by the λ-red-recombinase based method using the primers NK-*prc-*F and NK-*prc-*R, as described previously (Datsenko and Wanner, 2000;Wang et al., 2012). To construct double or single gene deletion mutant strains, the Keio collection, which is an *E. coli* BW25113 single gene mutant library constructed by Baba et al., was utilized (Baba et al., 2006). The *mltG* single mutant of BW25113 was reconstructed by transferring the *mltG* mutation locus in the *mltG* mutant strain from the Keio collection to the wild-type BW25113 through P1 phage transduction, as described previously (Thomason et al., 2007). Similarly, to construct double mutant strains in the *prc* mutant background, the second mutations were transferred from the corresponding mutant strains of the Keio collection to the *prc* mutant strain through P1 phage transduction.

To construct the BW25113 strains harboring *mepS*-*3×FLAG* at the chromosomal locus (BW25113-*mepS-3×FLAG*, *∆prc*-BW25113-*mepS-3×FLAG*, *∆mltG*-BW25113-*mepS-3×FLAG*, and *∆mltG∆prc*-BW25113-*mepS-3×FLAG*), the 3×FLAG-encoding sequence was fused to the 3’ end of *mepS* at its native chromosomal locus, followed by a chloramphenicol resistance cassette (Cm^r^). To do so, 3 PCR fragments, F1, Fragment 3×FLAG, and F2, were generated and then fused by PCR. The resulting final PCR products were used to replace the *mepS* gene in the chromosomal locus in the corresponding strains using the λ-red recombinase-based allelic exchange approach described previously (Datsenko and Wanner, 2000). Fragment F1 encoding the *mepS* sequence was generated with the primers WC107 and WC108 using the chromosomal DNA of BW25113 as the template. Fragment 3×FLAG encodes mainly 3×FLAG with its 5’ and 3’ ends homologous to the 3’ and 5’ ends of F1 and F2, respectively. This fragment was generated with the primers WC109 and WC110 using the plasmid pCA3×FLAG as the template. Fragment F2 encodes mainly a chloramphenicol resistance cassette flanked with a 5’ end sequence homologous to the 3’ end of Fragment 3×FLAG and with a 5’ end sequence homologous to the downstream sequence of *mepS* in the chromosome. The primers WC111 and WC102 were used to produce F2 using the pKD3 plasmid as the template for the Cm^r^. F1, Fragment 3×FLAG, and F2 were fused by PCR using WC107 and WC102. Similarly, to construct *Δprc*-BW25113-*lacZ::mltG-3×FLAG*, 5 PCR fragments were prepared and fused. The fragments are the upstream homologous fragment of lacZ (the primers WC112 and WC113), the fragment of the *mltG* promoter (the primers WC114 and WC115), the fragment containing *mltG* (the primers WC116 and WC117), the fragment encoding 3×FLAG fused with Cm^r^ (the primers WC118 and WC119), and the downstream homologous fragment of lacZ (the primers WC120 and WC121). The 3xFLAG-Cm^r^ fragment was amplified from the 3xFLAG-Cm^r^ sequence in BW25113-*mepS-3×FLAG*, while the other fragments were amplified from the chromosome of BW25113.

To construct the BW25113 strains harboring *mltG*-*HA* at the chromosomal locus (BW25113-MltG-HA, *∆prc*-BW25113-MltG-HA, BW25113-MltG-HA-MepS*-*3×FLAG, and *∆nlpI*-BW25113-MltG-HA-MepS*-*3×FLAG), the HA-encoding sequence was fused to the 3’ end of *mltG* at its native chromosomal locus, followed by a Cm^r^. To do so, 3 PCR fragments, F3, F4, and F5, were generated and then fused by PCR. The resulting final PCR products were used to replace the *mltG* gene in the chromosomal locus in WT-BW25113 using the λ-red recombinase-based allelic exchange approach described previously (Datsenko and Wanner, 2000), resulting in BW25113-MltG-HA . F3 contains the *mltG* sequence fused with a HA-encoding sequence at the 3’ end, which was generated with the primers WC103 and WC104 using the chromosomal DNA of BW25113 as the template. F4 encodes mainly a Cm^r^ with its 5’ and 3’ ends homologous to the 3’ and 5’ ends of F3 and F5, respectively. This fragment was generated with the primers WC101 and WC102 using the plasmid pKD3 as the template. F5 encodes mainly a downstream sequence of *mltG* in the chromosome flanked with a 5’ end sequence homologous to the 3’ end of Fragment F4. The primers WC105 and WC106 were used to produce F5 using the chromosomal DNA of BW25113 as the template. The fragments, F3, F4, and F5 were fused by PCR using WC103 and WC106. Then, the chromosomal locus of *mltG*, which was fused with a HA-encoding sequence and Cm^r^, in BW25113-MltG-HA was transferred to proper *E. coli* strains to produce *∆prc*-BW25113-MltG-HA, BW25113-MltG-HA-MepS*-*3×FLAG, and *∆nlpI*-BW25113-MltG-HA-MepS*-*3×FLAG.

**Plasmid construction**

To construct pBAD24-MltG-HA, the sequence encoding C-terminally HA-tagged MltG (*mltG*-HA) was produced by PCR with the primers WC122 and WC123. The PCR products were digested with EcoRI and HindIII and cloned into pBAD24. To construct pACYC184-MltG-HA, the *yecG*-HA fragment was PCR amplified from pBAD24-MltG-HA using the primers W124 and W125. Then, the pACYC184 plasmid was PCR amplified with the primers WC126 and WC127. The resulting fragment and *yecG*-HA were then fused by PCR with the primers WC125 and WC127. After NotI digestion, the fused fragment was self-ligated to become pACYC184-MltG-HA.

The plasmid pBAD24-His-MltG was constructed to express the recombinant MltG proteins with their signal sequence (the N-terminal 26 aa) replaced with a 6xHis tag. The *mltG* fragment was PCR amplified with the primers WC132 and WC133. After restriction digestion with EcoRI and HindIII, pBAD24 and the fragment were ligated to become pBAD24-His-MltG.

The plasmid pBAD24-MltG-His was constructed to express the C-terminally 6xHis-tagged MltG with a signal sequence (the N-terminal 26 aa) truncation. The *mltG* fragment was PCR amplified with the primers WC134 and WC135. After restriction digestion with EcoRI and HindIII, pBAD24 and the PCR product were ligated to become pBAD24-MltG-His.

To express the Prc variant ΔPDZ-K455A-Prc, the sequence encoding the PDZ domain was removed from the Prc gene of pTR163, which encoded K455A-Prc (Spiers et al., 2002). To do so, pTR163 was used as a template and amplified by using the primers WC130 and WC131. After digestion with EcoRI, the PCR product was self-ligated to become pTR163-∆PDZ, which encodes ΔPDZ-K455A-Prc.

**Table S1. The *E. coli* proteins showing stronger interaction signals with Prc or K455A-Prc than with ΔPDZ-K455A-Prc in the *E. coli* proteome array analyses**

| **Number** | **Protein** | **Gene** | **EcoGene ID** | **Functions** |
| --- | --- | --- | --- | --- |
| 1 | AmyA | *amyA* | EG11387 | alpha-amylase activity; calcium ion binding; carbohydrate metabolic process; |
| 2 | AtoB | *atoB* | EG11672 | acetyl-CoA C-acetyltransferase activity; fatty acid metabolic process; |
| 3 | AtoC | *atoC* | EG11668 | two-component response regulator activity; DNA binding; sequence-specific DNA binding transcription factor activity; ATP binding; transcription factor binding; nucleoside-triphosphatase activity; two-component signal transduction system (phosphorelay); transcription DNA-dependent; intracellular signal transduction; positive regulation of transcription DNA-dependent; |
| 4 | BglX | *bglX* | EG12013 | beta-glucosidase activity; carbohydrate metabolic process; |
| 5 | CdaR | *cdaR* | EG12335 | sequence-specific DNA binding transcription factor activity; transcription DNA-dependent; positive regulation of transcription DNA-dependent; |
| 6 | ChbB | *chbB* | EG10140 | protein-N(PI)-phosphohistidine-sugar phosphotransferase activity; kinase activity; phosphoenolpyruvate-dependent sugar phosphotransferase system; phosphorylation; |
| 7 | ChbF | *chbF* | EG10144 | nucleotide binding; 6-phospho-beta-glucosidase activity; oxidoreductase activity acting on the CH-OH group of donors NAD or NADP as acceptor; metal ion binding; carbohydrate catabolic process; oxidation-reduction process; |
| 8 | CitC | *citC* | EG13645 | ATP binding; N-acetyltransferase activity; [citrate (pro-3S)-lyase] ligase activity; biosynthetic process; |
| 9 | CyaY | *cyaY* | EG11653 | ferrous iron binding; ferric iron binding; protein complex assembly; iron-sulfur cluster assembly; |
| 10 | Dos | *dosP* | EG13792 | two-component sensor activity; magnesium ion binding; phosphorus-oxygen lyase activity; heme binding; cyclic-guanylate-specific phosphodiesterase activity; two-component signal transduction system (phosphorelay); transcription DNA-dependent; regulation of transcription DNA-dependent; cyclic nucleotide biosynthetic process; signal transduction by phosphorylation; intracellular signal transduction; |
| 11 | EutB | *eutB* | EG50006 | ethanolamine ammonia-lyase activity; cellular amino acid metabolic process; ethanolamine catabolic process; ethanolamine ammonia-lyase complex; |
| 12 | EutI | *eutD* | EG14188 | phosphate acetyltransferase activity; ethanolamine catabolic process; |
| 13 | EvgS | *evgS* | EG11610 | two-component sensor activity; two-component response regulator activity; transporter activity; ATP binding; histidine phosphotransfer kinase activity; two-component signal transduction system (phosphorelay); regulation of transcription DNA-dependent; transport; peptidyl-histidine phosphorylation; signal transduction by phosphorylation; intracellular signal transduction; protein autophosphorylation; intracellular; plasma membrane; |
| 14 | FolM | *folM* | EG13189 | nucleotide binding; dihydrofolate reductase activity; dihydromonapterin reductase activity; one-carbon metabolic process; 10-formyltetrahydrofolate biosynthetic process; folic acid biosynthetic process; oxidation-reduction process; |
| 15 | GabT | *gabT* | EG10361 | 4-aminobutyrate transaminase activity; pyridoxal phosphate binding; protein homodimerization activity; (S)-3-amino-2-methylpropionate transaminase activity; gamma-aminobutyric acid catabolic process; |
| 16 | GlnE | *glnE* | EG11602 | ATP binding; [glutamate-ammonia-ligase] adenylyltransferase activity; |
| 17 | GroL | *groL* | EG10599 | ATP binding; ATPase activity; identical protein binding; unfolded protein binding; ATP catabolic process; cell cycle; response to heat; protein refolding; phage assembly; cell division; |
| 18 | GspH | *gspH* | EG12887 | protein transporter activity; protein secretion by the type II secretion system; type II protein secretion system complex; |
| 19 | GuaD | *guaD* | EG13066 | zinc ion binding; guanine deaminase activity; ammeline aminohydrolase activity; guanine catabolic process; |
| 20 | HflC | *hflC* | EG10435 | peptidase activity; response to heat; negative regulation of metalloenzyme activity; |
| 21 | HisB | *hisB* | EG10445 | histidinol-phosphatase activity; imidazoleglycerol-phosphate dehydratase activity; histidine biosynthetic process; dephosphorylation; |
| 22 | HisP | *hisP* | EG10452 | ATP binding; ATPase activity; ATP catabolic process; histidine transport; |
| 23 | **HupA** | ***hupA*** | **EG10466** | **HU DNA-binding transcriptional dual regulator; DNA binding; protein binding; transcription DNA-dependent; response to DNA damage stimulus; chromosome condensation;** |
| 24 | InsB | *insB1* | EG40002 | DNA binding; transposase activity; transposition DNA-mediated; |
| 25 | KdpD | *kdpD* | EG10516 | two-component sensor activity; aspartate kinase activity; ATP binding; osmosensory signaling pathway via two-component system; detection of chemical stimulus; peptidyl-histidine phosphorylation; signal transduction by phosphorylation; protein autophosphorylation; |
| 26 | kptA | *kptA* | EG12570 | phosphotransferase activity alcohol group as acceptor; tRNA splicing via endonucleolytic cleavage and ligation; |
| 27 | LsrF | *lsrF* | EG13810 | aldehyde-lyase activity; metabolic process; |
| 28 | MalK | *malK* | EG10558 | ATP binding; maltose-transporting ATPase activity; maltooligosaccharide-importing ATPase activity; ATP catabolic process; maltose transport; maltodextrin transport; plasma membrane; ATP-binding cassette (ABC) transporter complex; |
| 29 | MalT | *malT* | EG10562 | two-component response regulator activity; sequence-specific DNA binding transcription factor activity; protein binding; ATP binding; sequence-specific DNA binding; trisaccharide binding; maltose catabolic process; two-component signal transduction system (phosphorelay); transcription DNA-dependent; regulation of transcription DNA-dependent; intracellular signal transduction; |
| 30 | MetR | *metR* | EG10591 | DNA binding; sequence-specific DNA binding transcription factor activity; transcription DNA-dependent; regulation of transcription DNA-dependent; methionine biosynthetic process; |
| 31 | MinC | *minC* | EG10596 | cell morphogenesis; barrier septum assembly; cell cycle; cell division; regulation of cell division; regulation of cell cycle; |
| 32 | Mqo | *mqo* | EG12069 | malate dehydrogenase (quinone) activity; malate dehydrogenase (menaquinone) activity; tricarboxylic acid cycle; oxidation-reduction process; |
| 33 | Mrp | *mrp* | EG10611 | ATP binding; |
| 34 | PaaG | *paaG* | EG13741 | lyase activity; isomerase activity; phenylacetate catabolic process; |
| 35 | PolB | *polB* | EG10747 | nucleotide binding; DNA binding; DNA-directed DNA polymerase activity; 3'-5'-exodeoxyribonuclease activity; DNA-dependent DNA replication; nucleotide-excision repair DNA gap filling; SOS response; translesion synthesis; DNA replication proofreading; |
| 36 | PssA | *pssA* | EG10781 | CDP-diacylglycerol-serine O-phosphatidyltransferase activity; phospholipid biosynthetic process; |
| 37 | PtsP | *ptsP* | EG12188 | sugar:hydrogen symporter activity; phosphoenolpyruvate-protein phosphotransferase activity; kinase activity; metal ion binding; phosphoenolpyruvate-dependent sugar phosphotransferase system; phosphorylation; |
| 38 | RecB | *recB* | EG10824 | DNA binding; ATP-dependent DNA helicase activity; endonuclease activity; protein binding; ATP binding; exodeoxyribonuclease V activity; double-strand break repair; DNA recombination; nucleic acid phosphodiester bond hydrolysis; exodeoxyribonuclease V complex; |
| 39 | RecC | *recC* | EG10825 | ATP-dependent DNA helicase activity; endonuclease activity; protein binding; ATP binding; exodeoxyribonuclease V activity; double-strand break repair; DNA recombination; nucleic acid phosphodiester bond hydrolysis; exodeoxyribonuclease V complex; |
| 40 | **RfaD** | ***rfaD*** | **EG10838** | **ADP-L-glycero-D-manno-heptose-6-epimerase; NADP+ binding; response to stress; lipopolysaccharide biosynthetic process; lipopolysaccharide core region biosynthetic process; ADP-L-glycero-beta-D-manno-heptose biosynthetic process;** |
| 41 | RplK | *rplK* | EG10872 | structural constituent of ribosome; protein binding; rRNA binding; translation; translational termination; stringent response; ribosome; cytosolic large ribosomal subunit; |
| 42 | RpsM | *rpsM* | EG10912 | tRNA binding; structural constituent of ribosome; rRNA binding; translation; ribosome biogenesis; intracellular; ribosome; cytosolic small ribosomal subunit; |
| 43 | RrmA | *rlmA* | EG12207 | zinc ion binding; 23S rRNA (guanine(745)-N(1))-methyltransferase activity; rRNA base methylation; |
| 44 | SelB | *selB* | EG10942 | tRNA binding; translation elongation factor activity; GTPase activity; GTP binding; GDP binding; selenocysteine insertion sequence binding; selenocysteine incorporation; GTP catabolic process; translation; selenocysteine metabolic process; |
| 45 | Slt | *slt* | EG10950 | hydrolase activity hydrolyzing O-glycosyl compounds; lytic transglycosylase activity; carbon-oxygen lyase activity acting on polysaccharides; peptidoglycan metabolic process; cellular cell wall organization; |
| 46 | SlyA | *slyA* | EG13408 | DNA binding; sequence-specific DNA binding transcription factor activity; transcription DNA-dependent; regulation of transcription DNA-dependent; pathogenesis; |
| 47 | TbpA | *thiB* | EG11574 | ATP binding; ATPase activity coupled to transmembrane movement of substances; transport; |
| 48 | TrpD | *trpD* | EG11027 | anthranilate phosphoribosyltransferase activity; anthranilate synthase activity; tryptophan biosynthetic process; glutamine metabolic process; |
| 49 | TruA | *truA* | EG10454 | tRNA binding; pseudouridine synthase activity; tRNA pseudouridine synthesis; |
| 50 | TynA | *tynA* | EG13140 | copper ion binding; calcium ion binding; primary amine oxidase activity; protein homodimerization activity; quinone binding; tryptamine:oxygen oxidoreductase (deaminating) activity; aminoacetone:oxygen oxidoreductase(deaminating) activity; aliphatic-amine oxidase activity; phenethylamine:oxygen oxidoreductase (deaminating) activity; L-phenylalanine catabolic process; phenylethylamine catabolic process; oxidation-reduction process; |
| 51 | **TyrA** | ***tyrA*** | **EG11039** | **Fused chorismate mutase/prephenate dehydrogenase; nucleotide binding; chorismate mutase activity; prephenate dehydrogenase (NADP+) activity; prephenate dehydrogenase activity; protein homodimerization activity; tyrosine biosynthetic process; L-phenylalanine biosynthetic process; chorismate metabolic process; oxidation-reduction process;** |
| 52 | XylB | *xylB* | EG11075 | xylulokinase activity; ATP binding; xylulose catabolic process; phosphorylation; D-xylose catabolic process; |
| 53 | **YaaW** | ***yaaW*** | **EG14340** | **unknown;** |
| 54 | **YacF** | ***zapD*** | **EG12313** | **cell divison; barrier septum assembly; cell cycle;** |
| 55 | YagR | *paoC* | EG13557 | xanthine dehydrogenase activity; protein binding; oxidoreductase activity acting on the aldehyde or oxo group of donors; molybdenum ion binding; purine nucleobase metabolic process; purine ribonucleoside salvage; response to DNA damage stimulus; oxidation-reduction process; |
| 56 | YahF | *yahF* | EG13590 | catalytic activity; cofactor binding; metabolic process; |
| 57 | YajL | *yajL* | EG13272 | protein homodimerization activity; thiamine biosynthetic process; cellular response to oxidative stress; protein refolding; ribosome biogenesis; |
| 58 | YbaJ | *tomB* | EG12429 | DNA binding; single-species biofilm formation; |
| 59 | YbcJ | *ybcJ* | EG12879 | RNA binding; |
| 60 | YbiA | *ybiA* | EG11579 | cellular component movement; |
| 61 | **MltG** | ***mltG*** | **EG11494** | **endolytic murein transglycosylase** |
| 62 | YcjM | *ycjM* | EG13910 | sucrose phosphorylase activity; cation binding; sucrose metabolic process; |
| 63 | **YdhS** | ***ydhS*** | **EG13953** | **unknown;** |
| 64 | YeaT | *dmlR* | EG13506 | DNA binding; sequence-specific DNA binding transcription factor activity; malate metabolic process; transcription DNA-dependent; negative regulation of transcription DNA-dependent; positive regulation of transcription DNA-dependent; |
| 65 | YeaU | *dmlA* | EG13507 | magnesium ion binding; isocitrate dehydrogenase (NADP+) activity; tartrate dehydrogenase activity; D-malate dehydrogenase (decarboxylating) activity; NAD binding; tricarboxylic acid cycle; malate metabolic process; oxidation-reduction process; |
| 66 | **YegV** | ***yegV*** | **EG14065** | **Putative sugar kinase; ribokinase activity; D-ribose metabolic process; phosphorylation;** |
| 67 | YejA | *yejA* | EG12037 | transporter activity; ATPase activity coupled to movement of substances; microcin transport; ATP-binding cassette (ABC) transporter complex; |
| 68 | **YfaU** | ***rhmA*** | **EG14083** | **2-keto-3-deoxy-L-rhamnonate aldolase; protein binding; nickel cation binding; aldehyde-lyase activity; cellular aromatic compound metabolic process;** |
| 69 | YfcH | *yfcH* | EG14111 | nucleotide binding; catalytic activity; coenzyme binding; cellular metabolic process; |
| 70 | YffB | *yffB* | EG11147 | unknown; |
| 71 | YfgC | *bepA* | EG14199 | metalloendopeptidase activity; proteolysis; |
| 72 | YfjK | *yfjK* | EG13197 | nucleic acid binding; ATP binding; ATP-dependent helicase activity; |
| 73 | YfjN | *rnlA* | EG13200 | endoribonuclease activity; protein binding; mRNA catabolic process; |
| 74 | YgaV | *ygaV* | EG13524 | DNA binding; sequence-specific DNA binding transcription factor activity; transcription DNA-dependent; regulation of transcription DNA-dependent; |
| 75 | YgcL | *casA* | EG13119 | RNA binding; defense response to virus; protein complex; |
| 76 | YgdH | *ygdH* | EG12373 | unknown; |
| 77 | YgfY | *sdhE* | EG13075 | negative regulation of molecular function; |
| 78 | YhcO | *yhcO* | EG12821 | unknown; |
| 79 | YhgF | *yhgF* | EG12932 | RNA binding; hydrolase activity acting on ester bonds; nucleobase-containing compound metabolic process; |
| 80 | **YidA** | ***yidA*** | **EG11195** | **sugar-phosphatase activity; magnesium ion binding; dephosphorylation;** |
| 81 | YjdA | *crfC* | EG11210 | DNA binding; GTPase activity; ATP binding; GTP binding; GTP catabolic process; organic phosphonate transport; |
| 82 | YjgB | *ahr* | EG11436 | nucleotide binding; zinc ion binding; oxidoreductase activity acting on the CH-OH group of donors NAD or NADP as acceptor; cofactor binding; oxidation-reduction process; |
| 83 | YmfT | *ymfT* | EG14414 | DNA binding; |
| 84 | YnfO | *ynfO* | EG14422 | unknown; |
| 85 | YpfI | *tmcA* | EG14196 | tRNA binding; ATP binding; tRNA N-acetyltransferase activity; tRNA wobble cytosine modification; tRNA acetylation; |

Note: Proteins (genes) shown in bold were demonstrated to be Prc-cleavable in Vitro.

**Table S2. The C-terminal residues of Prc substrates**

| Prc substrates | ^a^The C-terminal 20 amino acid residues of the Prc substrates | Reference |
| --- | --- | --- |
| MltG | A**SHNKS**V**QD**YL**K**VL**KEKN**A**Q** | This study |
| MepS | M**NE**P**Y**W**KKRYNE**A**RR**VL**SRS** | (Singh et al., 2015) |
| PBP3 | **TT**G**DKNEF**VI**NQ**G**E**G**T**GG**RS** | (Nagasawa et al., 1989) |
| β-casein | FLL**YQE**PVLGPV**R**GPFPIIV |  |
| GST-SsrA^b^ | FGGG**DH**PP**K**AA**NDENT**ALAA^c^ | (Karzai et al., 2000) |
| λR-WVAAA^d^ | **E**I**YE**M**YE**AV**S**M**Q**P**S**L**W**VAAA^e^ | (Parsell et al., 1990) |

^a^ Polar amino acids are shown in bold.

^b^GST-SsrA, Glutathione S-transferase (GST) C-terminally fused with the SsrA peptide.

^c^ The SsrA peptide sequence is marked by underline.

^d^λR-WVAAA, N-terminal λ-repressor (residues 1-102) with its C-terminal 5 residues replaced　with WVAAA.

^e^ The N-terminal λ-repressor sequence is marked by underline.

**Table S3. Primers used in this study**

| **Names** | **Sequences (5'→3')** |
| --- | --- |
| **WC101** | cagTACCCATACGATGTTCCAGATTACGCTtaaataggaatatcctccttagt |
| **WC102** | CTCGTCAGGATAGCCAAGGGATTGCATCCAAACGGTTTATGTGTAGGCTGGAGCTGCTTC |
| **WC103** | Tgctggtggtactgggtatc |
| **WC104** | TTAAGCGTAATCTGGAACATCGTATGGGTACTGCGCATTTTTTTCCTTAAG |
| **WC105** | Tggatgcaatcccttggctatcctgacgagagtctgtgcaggattatctg |
| **WC106** | TGCTCAATACGATCCAGCTC |
| **WC107** | ATGGTCAAATCTCAACCG |
| **WC108** | GCTGCGGCTGAGAACCCG |
| **WC109** | GAAGCGTTACAACGAAGCACGCCGGGTTCTCAGCCGCAGCGACTACAAAGACCATGACG |
| **WC110** | CGGAATAGGAACTAAGGAGGATATTCCTATGTAAAACGACGGCCAGTGAATTG |
| **WC111** | ATAGGAATATCCTCCTTAGTTC |
| **WC112** | CCGGAAGAGAGTCAATTCAG |
| **WC113** | CATATGATAAGCATCGCTGCTCATACCAAAGCCGTTGATGGGTGTCTGGTCAG |
| **WC114** | GGCTTTGGTATGAGCAGCGATGCTTATCATATG |
| **WC115** | AAGTGGGGCTAAATATTCATATAACGTTGCGACTTTCAGCACACCATTTTG |
| **WC116** | GCAACGTTATATGAATATTTAGCCCCACTTTG |
| **WC117** | CTTTATAATCACCGTCATGGTCTTTGTAGTCCTGCGCATTTTTTTCCTTAAG |
| **WC118** | GACTACAAAGACCATGACG |
| **WC119** | TGTGTAGGCTGGAGCTGCTT |
| **WC120** | AAGCAGCTCCAGCCTACACAGAGCTCCTGCACTGGATGGTG |
| **WC121** | AGACCAACTGGTAATGGTAG |
| **WC122** | GGAGGAATTCACCATGAAAAAAGTGTTATTGATAATC |
| **WC123** | CAGCCAAGCTTAAGCGTAATCTGGAACATCGTATGGGTACTGCGCATTTTTTTCCTTAAG |
| **WC124** | TCGAGATTTTCAGGAGCTAAGGAAGCTAAAATGAAAAAAGTGTTATTGATAATC |
| **WC125** | ATATGCGGCCGCTTAAGCGTAATCTGGAACATCGTATGGGTAC |
| **WC126** | TTTAGCTTCCTTAGCTCCTG |
| **WC127** | AGTCGCGGCCGCATACGCAAGGCGACAAGGTG |
| **WC130** | TGGTGAATTCAAAGGGACCAAGACCCGTAC |
| **WC131** | GATCGAATTCGTCCATGGTCTGTTTCCTGTG |
| **WC132** | ATGGGAATTCACCATGCACCACCACCACCACCACCGCCATCTTGCCGACAGCAAATTG |
| **WC133** | GCCCAAGCTTTTACTGCGCATTTTTTTCCTTAAG |
| **WC134** | GGAGGAATTCACCATGCGCCATCTTGCCGACAGCAAATTGC |
| **WC135** | AGCCAAGCTTTTAGTGGTGGTGGTGGTGGTGCTGCGCATTTTTTTCCTTAAG |
| **NK-*prc-*F** | AGGCTTACCGCGTTAGCTGGCCTGCTTGCAATAGCAGGCCAGACCTTCGCCATATGAATATCCTCCTTAG |
| **NK-*prc-*R** | CAAGCTTCGCCAGATCGAGTGCGATATTCACCGTCTCATCCAGATAAGGAGTGTAGGCTGGAGCTGCTTC |

**References**

Baba, T., Ara, T., Hasegawa, M., Takai, Y., Okumura, Y., Baba, M., Datsenko, K.A., Tomita, M., Wanner, B.L., and Mori, H. (2006). Construction of Escherichia coli K-12 in-frame, single-gene knockout mutants: the Keio collection. *Mol Syst Biol* 2**,** 2006 0008.

Datsenko, K.A., and Wanner, B.L. (2000). One-step inactivation of chromosomal genes in Escherichia coli K-12 using PCR products. *Proc Natl Acad Sci U S A* 97**,** 6640-6645.

Karzai, A.W., Roche, E.D., and Sauer, R.T. (2000). The SsrA-SmpB system for protein tagging, directed degradation and ribosome rescue. *Nat Struct Biol* 7**,** 449-455.

Nagasawa, H., Sakagami, Y., Suzuki, A., Suzuki, H., Hara, H., and Hirota, Y. (1989). Determination of the cleavage site involved in C-terminal processing of penicillin-binding protein 3 of Escherichia coli. *J Bacteriol* 171**,** 5890-5893.

Parsell, D.A., Silber, K.R., and Sauer, R.T. (1990). Carboxy-terminal determinants of intracellular protein degradation. *Genes Dev* 4**,** 277-286.

Singh, S.K., Parveen, S., Saisree, L., and Reddy, M. (2015). Regulated proteolysis of a cross-link-specific peptidoglycan hydrolase contributes to bacterial morphogenesis. *Proc Natl Acad Sci U S A* 112**,** 10956-10961.

Spiers, A., Lamb, H.K., Cocklin, S., Wheeler, K.A., Budworth, J., Dodds, A.L., Pallen, M.J., Maskell, D.J., Charles, I.G., and Hawkins, A.R. (2002). PDZ domains facilitate binding of high temperature requirement protease A (HtrA) and tail-specific protease (Tsp) to heterologous substrates through recognition of the small stable RNA A (ssrA)-encoded peptide. *J Biol Chem* 277**,** 39443-39449.

Thomason, L.C., Costantino, N., and Court, D.L. (2007). E. coli genome manipulation by P1 transduction. *Curr Protoc Mol Biol* Chapter 1**,** Unit 1.17.

Wang, C.Y., Wang, S.W., Huang, W.C., Kim, K.S., Chang, N.S., Wang, Y.H., Wu, M.H., and Teng, C.H. (2012). Prc contributes to Escherichia coli evasion of classical complement-mediated serum killing. *Infect Immun* 80**,** 3399-3409.
